# Vitamin D ameliorates adipose browning in chronic kidney disease cachexia

**DOI:** 10.1038/s41598-020-70190-z

**Published:** 2020-08-25

**Authors:** Wai W. Cheung, Wei Ding, Hal M. Hoffman, Zhen Wang, Sheng Hao, Ronghao Zheng, Alex Gonzalez, Jian-Ying Zhan, Ping Zhou, Shiping Li, Mary C. Esparza, Richard L. Lieber, Robert H. Mak

**Affiliations:** 1 Pediatric Nephrology, Rady Children’s Hospital San Diego, University of California, San Diego, USA; 2 Division of Nephrology, School of Medicine, Shanghai Ninth People’s Hospital, Shanghai Jiaotong University, Shanghai, China; 3 Department of Pediatrics, University of California, San Diego, USA; 4 Department of Pediatrics, Shanghai General Hospital, Shanghai Jiao Tong University, Shanghai, China; 5 Department of Nephrology and Rheumatology, Shanghai Children’s Hospital, Shanghai Jiao Tong University, Shanghai, China; 6 Department of Pediatrics, Hubei Maternal and Child Health Hospital, Wuhan, China; 7 Children’s Hospital, Zhejiang University, Hangzhou, China; 8 Department of Pediatrics, The Second Affiliated Hospital of Harbin Medical University, Harbin, China; 9 College of Bioscience and Biotechnology, Yangzhou University, Yangzhou, China; 10 Department of Orthopedic Surgery, University of California, San Diego, USA; 11 Shirley Ryan AbilityLab and Northwestern University, Chicago, USA; 12 Division of Pediatric Nephrology, Department of Pediatrics, University of California, San Diego, 9500 Gilman Drive, MC 0831, La Jolla, CA 92093-0831 USA

**Keywords:** Molecular biology, Nephrology, Kidney

## Abstract

Patients with chronic kidney disease (CKD) are often 25(OH)D_3_ and 1,25(OH)_2_D_3_ insufficient. We studied whether vitamin D repletion could correct aberrant adipose tissue and muscle metabolism in a mouse model of CKD-associated cachexia. Intraperitoneal administration of 25(OH)D_3_ and 1,25(OH)_2_D_3_ (75 μg/kg/day and 60 ng/kg/day respectively for 6 weeks) normalized serum concentrations of 25(OH)D_3_ and 1,25(OH)_2_D_3_ in CKD mice. Vitamin D repletion stimulated appetite, normalized weight gain, and improved fat and lean mass content in CKD mice. Vitamin D supplementation attenuated expression of key molecules involved in adipose tissue browning and ameliorated expression of thermogenic genes in adipose tissue and skeletal muscle in CKD mice. Furthermore, repletion of vitamin D improved skeletal muscle fiber size and in vivo muscle function, normalized muscle collagen content and attenuated muscle fat infiltration as well as pathogenetic molecular pathways related to muscle mass regulation in CKD mice. RNAseq analysis was performed on the gastrocnemius muscle. Ingenuity Pathway Analysis revealed that the top 12 differentially expressed genes in CKD were correlated with impaired muscle and neuron regeneration, enhanced muscle thermogenesis and fibrosis. Importantly, vitamin D repletion normalized the expression of those 12 genes in CKD mice. Vitamin D repletion may be an effective therapeutic strategy for adipose tissue browning and muscle wasting in CKD patients.

## Introduction

Chronic kidney disease (CKD)-associated cachexia is a complex metabolic disorder that consists of anorexia, weight loss, loss of adipose tissue and muscle mass as well as hypermetabolism^1,2^. Current therapies focus on palliation, but calorie supplementation alone is not successful in treating CKD-associated cachexia^3^. Brown adipocytes and beige adipocytes, which reside within white adipose tissue (WAT), significantly contribute to whole body energy expenditure^4^. Beige adipocytes respond to cold stimulation in a process described as WAT browning^5^. We and others have demonstrated the presence of WAT browning in CKD mice^5^ amongst other animal disease models of cachexia as well as in patients with cachexia^6–9^. We also demonstrated WAT browning in a mouse model of cystinosis, a genetic cause of CKD^10,11^.

CKD patients have a high prevalence of 25-hydroxyvitamin D3 and 1,25-dihydroxyvitamin D3 insufficiency^12–14^. Vitamin D insufficiency^2,12^ may be an important cause of CKD-associated cachexia. Vitamin D influences myogenesis and muscle function^15,16^. Furthermore, vitamin D insufficiency has been correlated with reduced muscle size and strength in the general population. Supplementation of vitamin D was associated with increased muscle size and strength in patients on hemodialysis^17^. Vitamin D receptor (VDR) is expressed in skeletal muscle of mice and regulates uptake of 25-hydroxyvitamin D3 in myofibers^18^. Vitamin D exerts its effects via genomic and non-genomic, membrane-associated rapid response actions to regulate the function in muscle^19,20^. 25-hydroxyvitamin D3 could also directly exert paracrine and autocrine effects on muscle^15^. Low serum concentrations of 25-hydroxyvitamin D3 are an independent risk factor for decreased muscle function in elderly patients on dialysis^21^. Adipose tissue is an important storage site for vitamin D. Vitamin D insufficiency has been associated with aberrant adipogenesis^22^. There are no published data that report the direct impact of vitamin D repletion on muscle mass and adipose tissue metabolism in CKD mice. Thus, the purpose of this study was to investigate the impact of vitamin D repletion in a mouse model of CKD-associated cachexia.

## Results

### Vitamin D repletion normalizes serum vitamin D concentrations and improves energy homeostasis in CKD mice

**Figure 1:**
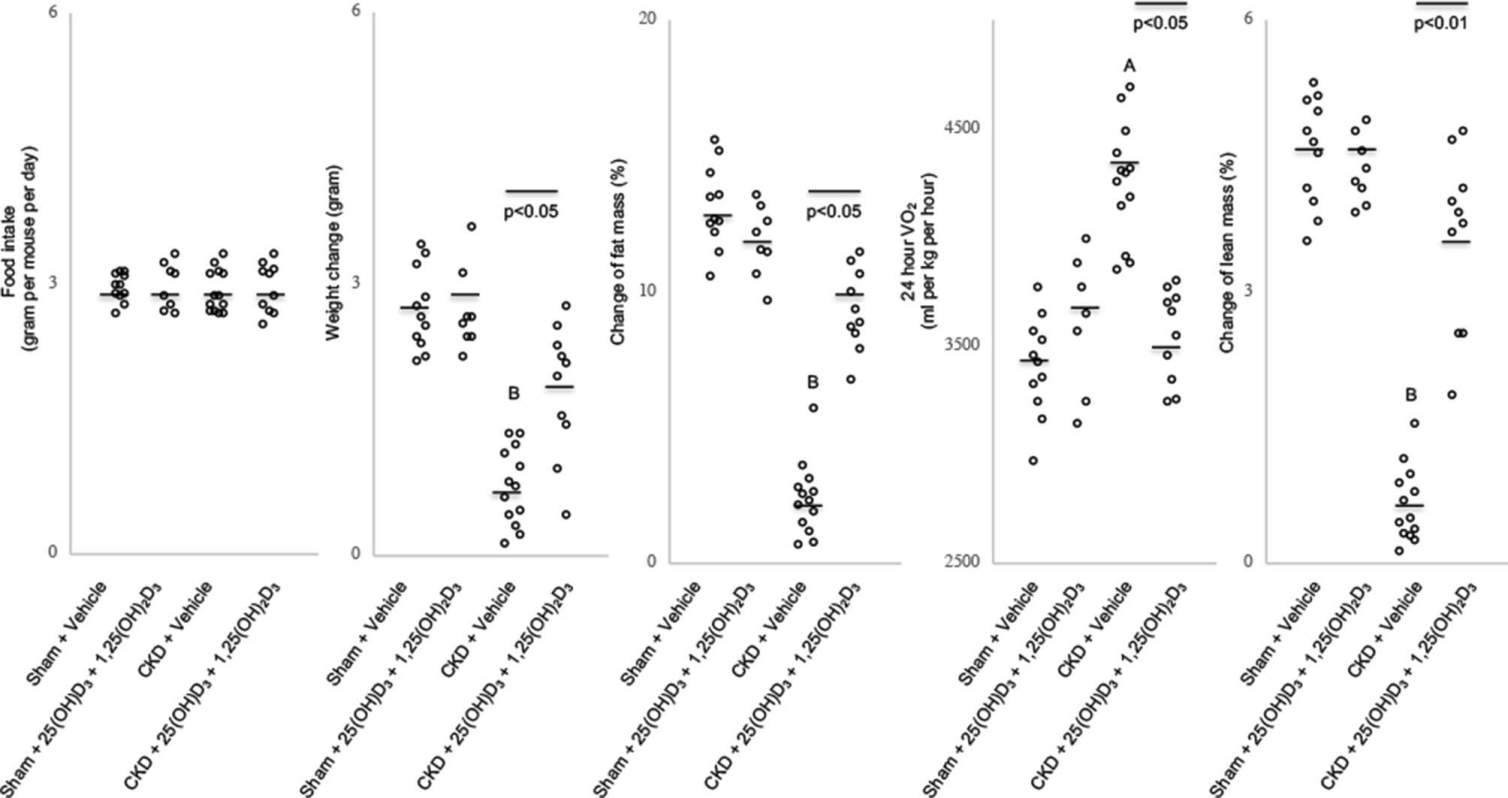
Supplementation of vitamin D ameliorates cachexia in CKD mice. Sham and CKD mice were treated with 25(OH)D_3_ and 1,25(OH)_2_D_3_ (75 μg/kg per day and 60 ng/kg per day, respectively) or ethylene glycol as vehicle for 6 weeks. Four groups of mice were included: Sham + Vehicle (n = 11), Sham + 25(OH)D_3_ + 1,25(OH)_2_D_3_ (n = 8), CKD + Vehicle (n = 13) and CKD + 25(OH)D_3_ + 1,25(OH)_2_D_3_ (n = 10). Data are expressed as mean ± SEM. ^A^ *p* < 0.05, significantly higher in CKD + Vehicle and CKD + 25(OH)D_3_+1,25(OH)_2_D_3_ mice versus Sham + Vehicle and Sham + 25(OH)D_3_ + 1,25(OH)_2_D_3_ mice, respectively. ^B^ *p* < 0.05, significantly lower in CKD + Vehicle and CKD + 25(OH)D_3_ + 1,25(OH)_2_D_3_ mice versus Sham + Vehicle and Sham + 25(OH)D_3_ + 1,25(OH)_2_D_3_ mice, respectively. Results of CKD + Vehicle mice were also compared to CKD + 25(OH)D_3_ + 1,25(OH)_2_D_3_ mice.

Serum concentrations of 25-hydroxyvitamin D3 and 1,25-dihydroxyvitamin D3 were significantly decreased in CKD mice compared to sham (Supplemental Table 1S). Supplementation of 25-hydroxyvitamin D3 and 1,25-dihydroxyvitamin D3 (75 μg/kg/day and 60 ng/kg/day, respectively, for 6 weeks) normalized serum vitamin D concentrations in CKD mice. Vitamin D repletion normalized food intake and improved body weight gain in CKD mice (Supplemental Fig. 1S). We further evaluated the effect of vitamin D repletion beyond appetite stimulation and resultant body weight gain in CKD mice by employing a pair-feeding strategy (Supplemental Fig. 2S). CKD mice and sham control mice were treated with 25-hydroxyvitamin D3 and 1,25-dihydroxyvitamin D3 or with vehicle, respectively. Vehicle treated CKD were fed ad libitum while other groups of mice were given the same amount of diet as vehicle treated CKD mice (Fig. 1). Results of serum chemistry of CKD mice after 6 weeks of vitamin D repletion or vehicle are shown (Table 1). Serum concentration of vitamin D binding protein (VDBP) was elevated in CKD mice versus sham mice. Repletion of vitamin D in CKD mice did not change serum VDBP levels. Circulating PTH levels were increased in CKD mice versus sham mice. Repletion of vitamin D decreased but did not normalize serum PTH levels in CKD mice. Importantly, repletion of vitamin D normalized the CKD cachexia phenotype including decreased weight gain, reduced fat mass content, elevated basal metabolic rate, and decreased lean mass (Fig. 1).

**Table 1:** Serum and blood chemistry of mice.

	Sham + Vehicle	Sham + 25(OH)D_3_ + 1,25(OH)_2_D_3_	CKD + Vehicle	CKD + 25(OH)D_3_ + 1,25(OH)_2_D_3_
	n = 11	n = 8	n = 13	n = 10
BUN (mg/dl)	27.7 ± 2.5	26.9 ± 2.9	79.6 ± 7.6 ^A^	80.2 ± 11.4 ^A^
Ca (mg/dl)	11.3 ± 0.8	11.3 ± 1.1	9.3 ± 0.5 ^B^	9.3 ± 0.7 ^B^
Creatinine (mg/dl)	< 0.2	< 0.2	0.7 ± 0.2 ^A^	0.8 ± 0.2 ^A^
Bicarbonate (mmol/l)	27.8 ± 5.7	27.8 ± 2.2	26.8 ± 3.7	28.4 ± 1.8
25(OH)D_3_ (ng/ml)	107.9 ± 12.4	112.4 ± 13.2	43.2 ± 10.3 ^B,C^	97.7 ± 8.9
1,25(OH)_2_D_3_ (pg/ml)	274.3 ± 32.1	259.8 ± 21.4	104.3 ± 19.4 ^B,C^	221.4 ± 15.8
Pi (mg/dl)	7.3 ± 0.3	7.5 ± 0.3	9.5 ± 0.3 ^A^	9.3 ± 0.5 ^A^
PTH (pg/ml)	119.7 ± 23.6	102.1 ± 6.7	325.7 ± 21.7 ^A,C^	216.2 ± 17.4 ^A^
VDBP (μg/ml)	396.3 ± 34.5	492.1 ± 25.4	628.3 ± 22.4 ^A^	714.9 ± 31.5 ^A^

Sham and CKD mice were treated with 25(OH)D_3_ and 1,25(OH)_2_D_3_ (75 μg/kg per day and 60 ng/kg per day, respectively) or ethylene glycol as vehicle for 6 weeks. Data are expressed as mean ± SEM. ^A^*p* < 0.05, significantly higher in CKD + Vehicle and CKD + 25(OH)D_3_ + 1,25(OH)_2_D_3_ mice versus Sham + Vehicle and Sham + 25(OH)D_3_ + 1,25(OH)_2_D_3_ mice, respectively. ^B^*p* < 0.05, significantly lower in CKD + Vehicle and CKD + 25(OH)D_3_ + 1,25(OH)_2_D_3_ mice versus Sham + Vehicle and Sham + 25(OH)D_3_ + 1,25(OH)_2_D_3_ mice, respectively. ^C^*p* < 0.05, significantly different between CKD + Vehicle and CKD + 25(OH)D_3_ + 1,25(OH)_2_D_3_ mice.

### Vitamin D repletion normalizes uncoupling proteins and ATP contents as well as normalizes thermogenic gene expression in adipose tissue and muscle in CKD mice

**Figure 2:**
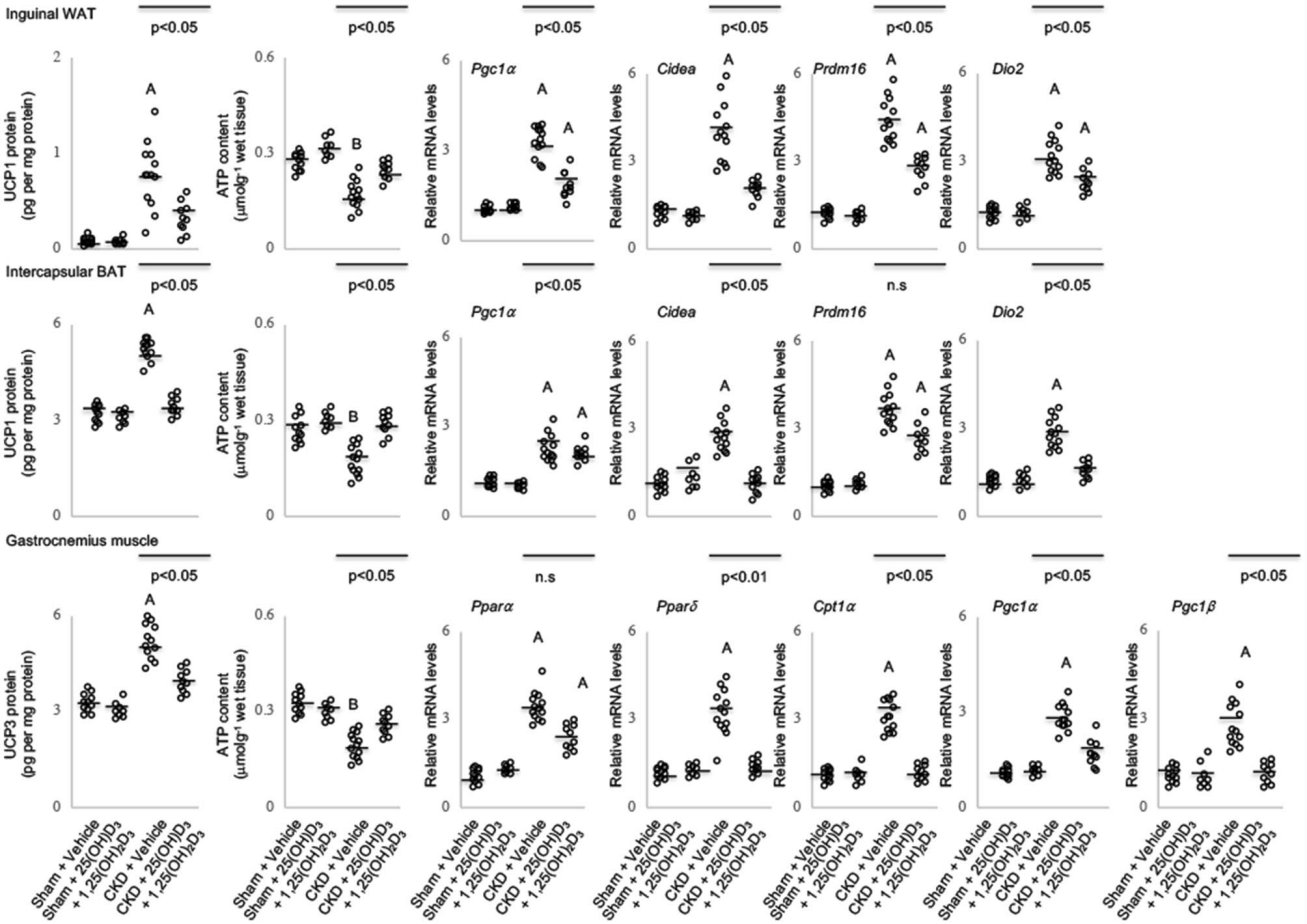
Supplementation of vitamin D modulates contents of uncoupling proteins and adenosine triphosphate as well as attenuates aberrant thermogenic gene expression in adipose tissue and skeletal muscle CKD mice. UCP and ATP content in inguinal white adipose tissue, brown adipose tissue and gastrocnemius muscle were measured. In addition, thermogenic gene expression (Pgc1α, Cidea, Prdm16 and Dio2) in inguinal white adipose tissue, brown adipose tissue as well as expression of genes related to fatty acid oxidation (Pparα, Pparδ and Cpt1α) and energy consumption (Pgc1α and Pgc1β) were measured in skeletal muscle in mice. Results are analyzed and expressed as in Fig. 1.

Adipose and muscle protein contents of UCPs were higher in CKD mice compared to sham mice. In contrast, adipose and muscle ATP contents were decreased in CKD mice compared to sham mice (Fig. 2). Repletion of vitamin D normalized UCPs and ATP content in adipose and muscle tissue in vehicle treated CKD mice versus vehicle treated sham mice. Expression of adipose tissue thermogenic genes (Pgc1*α*, Cidea, Prdm16 and Dio2) and skeletal muscle thermogenic genes (Ppar*α*, Ppar*δ*, Cpt1*α*, Pgc1*α* and Pgc1*β*) were significantly increased in CKD mice (Fig. 2). Vitamin D repletion ameliorated elevated adipose and muscle tissue thermogenic gene expression in CKD mice.

### Repletion of vitamin D attenuates adipose tissue browning in CKD mice

**Figure 3:**
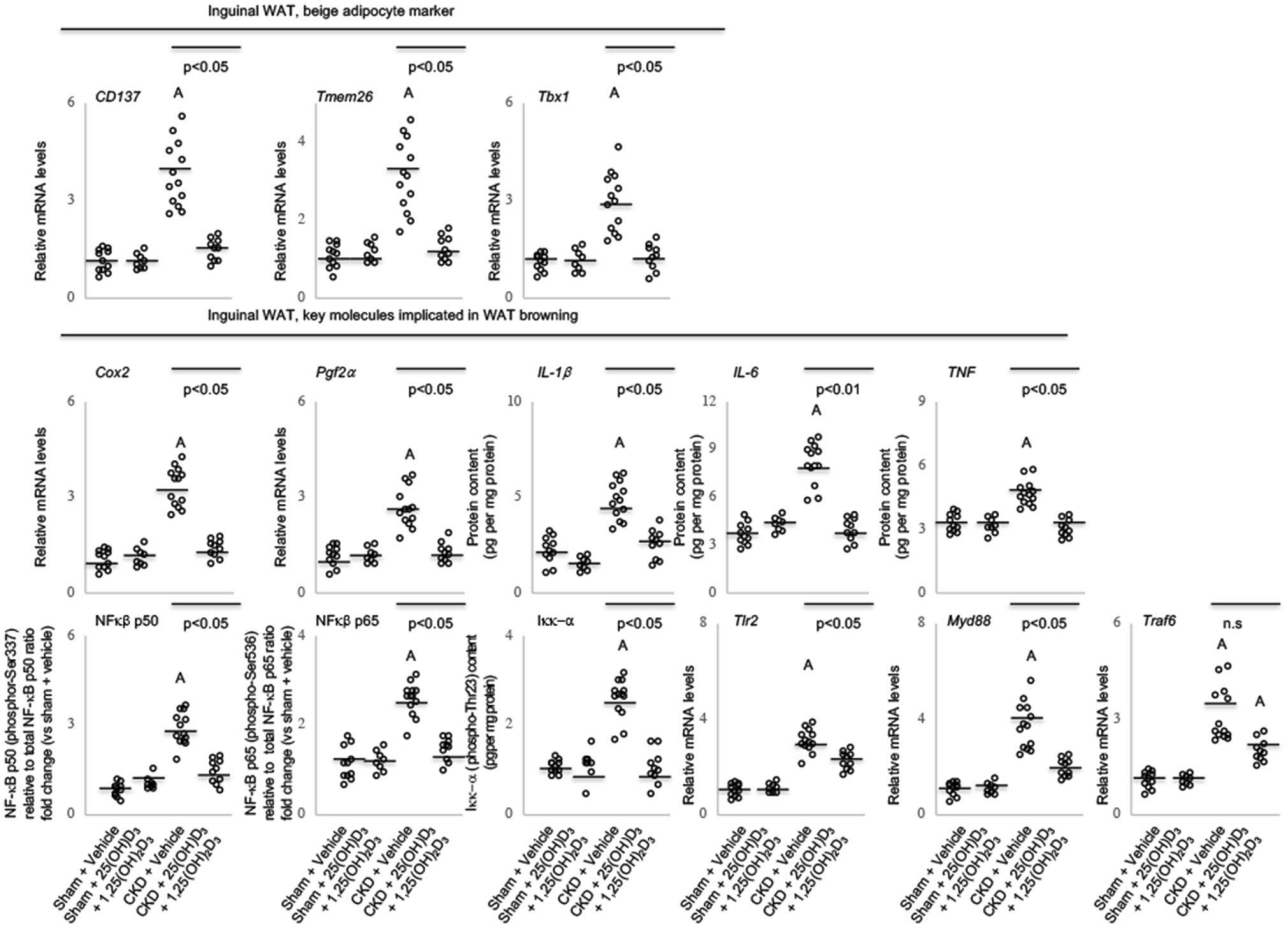
Vitamin D repletion attenuates adipose tissue browning in CKD mice. Gene expression of beige adipocyte markers (CD137, Tmen 26 and Tbx-1) in inguinal white adipose tissue was measured. We also measured gene expression of key molecules involved in adipose tissue browning in CKD mice. Gene expression of Cox2 signaling pathway (Cox2 and Pgf2α), protein content of inflammatory cytokines (IL-1α, IL-6, and TNF), relative phosphorylated NF-κB p50 (Ser337) / total p50 ratio, relative phosphorylated NF-κB p65 (Ser536) / total p65 ratio and phosphorylated Iκκ-a (Thr23) protein content as well as mRNA expression of Tlr pathway (Tlr2, Myd88 and Traf6) in inguinal white adipose tissue was measured. Results are analyzed and expressed as in Fig. 1.

WAT browning occurred in CKD mice based on the presence of beige adipocyte cell markers such as the expression of thermogenic UCP-1 protein content in inguinal WAT that is normally restricted to BAT (Fig. 2). In addition, increased expression of beige adipose cell surface markers (CD137, Tmem26 and Tbx1) was measured in inguinal WAT in CKD mice (Fig. 3). Repletion of vitamin D attenuated adipose tissue browning in CKD mice. Supplementation of vitamin D normalized protein content of UCP-1 and gene expression of beige adipose cell surface markers in inguinal WAT in CKD mice (Figs. 2 and 3). Cox2/Pgf2*α* and inflammatory cytokines stimulate beige adipogenesis. We showed that expression of Cox2/Pgf2*α* and protein content of IL-1*β*, IL-6 and TNF were significantly increased in inguinal WAT in CKD mice versus control mice (Fig. 3). The toll-like receptor (Tlr)/NF-*k*B signaling pathway modulates expression of inflammatory cytokines. We show that phosphor-NF-*k*B p50, phosphor-NF-*k*B p65 and phosphor-I*kk*-*a* protein content was significantly increased in inguinal WAT of CKD mice relative to sham mice (Fig. 3). Expression of Tlr2, myeloid differentiation primary response 88 (MyD88) and Traf6 were significantly increased in inguinal WAT in CKD mice relative to sham mice. We demonstrate that vitamin D repletion ameliorated expression of Cox2/Pgf2*α* as well as expression and protein content of key Tlr/NF-*k*B pathway molecules in inguinal WAT CKD mice relative to controls.

### Vitamin D repletion improves muscle fiber size and in vivo muscle function in CKD mice

**Figure 4:**
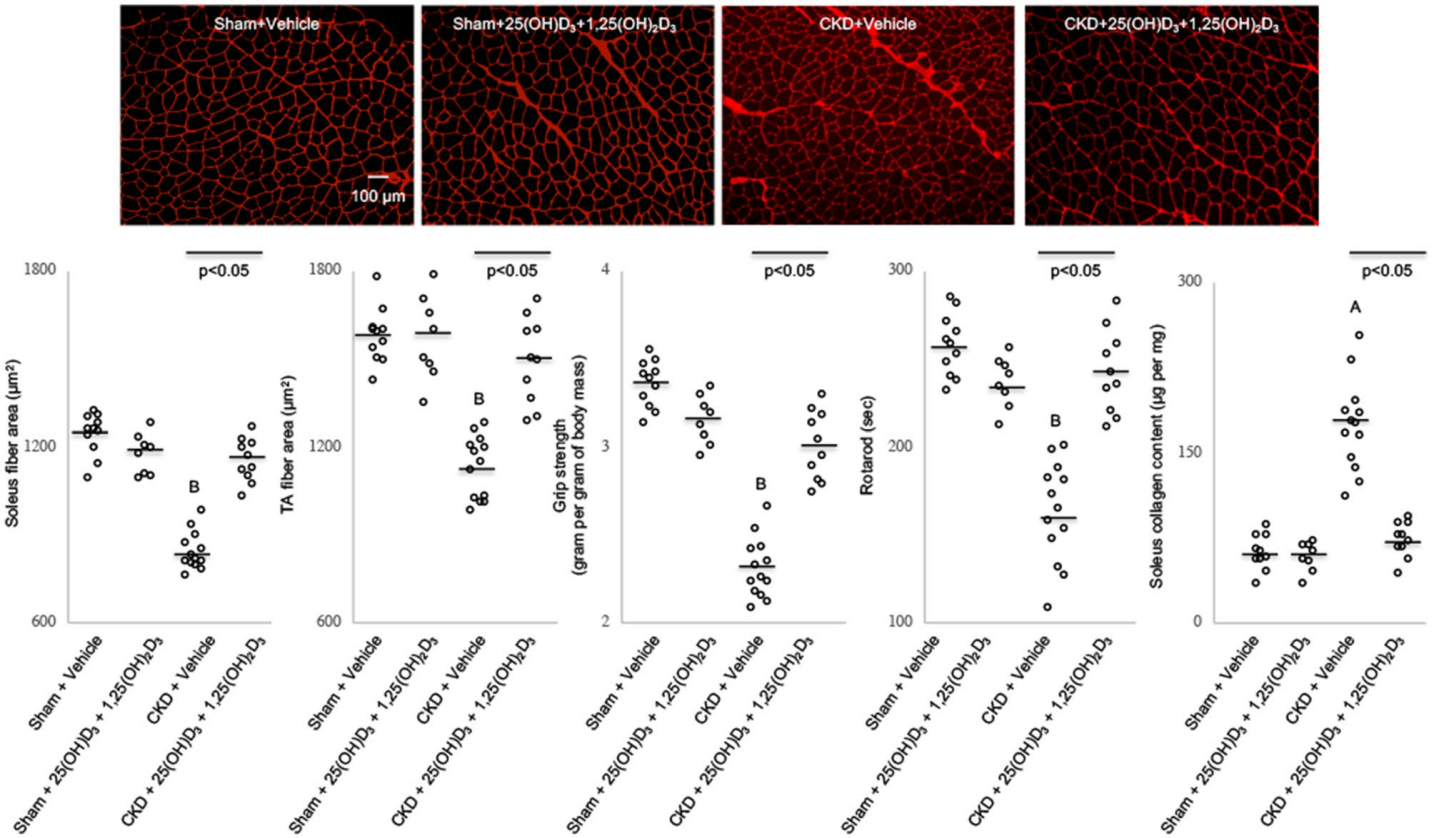
Vitamin D repletion improves muscle fiber size and in vivo muscle function as well as normalizes muscle collagen content in CKD mice. Representative photomicrographs of soleus immunohistochemical sections labeled with a polyclonal antibody to laminin with magnification ×200**.** Average soleus and tibialis anterior cross-sectional area as well as in vivo grip strength and rotarod activity were measured. Muscle collagen content was quantitated. Results are analyzed and expressed as in Fig. 1.

We evaluated the effect of vitamin D repletion on skeletal muscle morphology in CKD mice. Representative results of muscle sections are illustrated (Fig. 4). Repletion of vitamin D normalized average cross-sectional area of soleus and tibias anterior muscle in CKD mice. Patients with CKD are often afflicted with neuromuscular complications. We measured motor coordination in vitamin D and vehicle treated CKD mice. Vitamin D repletion normalized grip strength and rotarod fall latency in CKD mice.

### Vitamin D repletion decreases muscle pro-fibrotic and increases anti-fibrotic gene expression in CKD mice

Vitamin D repletion normalized soleus collagen content in CKD mice (Fig. 4). In addition, we measured gastrocnemius muscle expression of 84 key genes involved in tissue fibrosis in mice (Supplemental Table 2S). Pro-fibrotic genes (TGFα1, PAI-1, TGIF1, IL-1α, IL-1β, Agt, CTGF, Akt1, Smad3 and Timp3) were upregulated while anti-fibrotic genes (BMP7 and IL13R*α*2) were downregulated in CKD versus sham mice (Table 2). Importantly, repletion of vitamin D attenuated aberrant muscle expression of fibrotic genes in CKD mice (Table 3).

**Table 2:** Repletion of vitamin D modulates muscle fibrotic gene expression in CKD mice.

(A) Gene	Cell function	Fold, up-regulation	*p* value
TGFβ1	signal transduction, TGFβ superfamily	8.19 ± 0.64	0.001
PAI-1	ECM remodeling	8.09 ± 1.08	0.046
TGIF1	signal transduction, TGFβ superfamily	7.85 ± 2.24	0.002
IL-1α	inflammatory cytokines and chemokines	6.77 ± 0.72	0.022
IL-1β	inflammatory cytokines and chemokines	5.60 ± 0.52	0.004
Agt	pro-fibrotic, growth factor	5.12 ± 0.84	0.000
CTGF	pro-fibrotic	3.22 ± 1.56	0.037
Akt1	signal transduction, TGFβ superfamily	2.23 ± 0.26	0.040
Smad3	signal transduction, TGFβ superfamily	2.14 ± 0.70	0.016
Timp3	ECM remodeling	1.71 ± 0.48	0.037

(B) Gene	Cell function	Fold, down-regulation	*p* value
BMP7	Anti-fibrotic	(−) 4.82 ± 2.26	0.006
IL13Rα2	Anti-fibrotic	(−) 2.65 ± 1.14	0.005

Gastrocnemius muscle was used for differential fibrosis gene profiling using Qiagen PCR Array Mouse Fibrosis Catalog no. 330231 PAMM-120ZA. Muscle expression of a total of 84 genes important for fibrosis was compared between CKD mice (n = 4) and sham control mice (n = 4). Detailed information for 84 genes is listed in Supplemental Table 2S. Data are expressed as mean ± SEM (Table 2A). Subsequently, sham and CKD mice were treated with 25(OH)D_3_ and 1,25(OH)_2_D_3_ (75 μg/kg per day and 60 ng/kg per day, respectively) or ethylene glycol as vehicle for 6 weeks. Gastrocnemius muscle was used for mRNA expression study. Data are expressed and analyzed as in Table 1.

**Table 3:** Repletion of vitamin D modulates muscle fibrotic gene expression in CKD mice.

	Sham + Vehicle	Sham + 25(OH)D_3_ + 1,25(OH)_2_D_3_ n = 8	CKD + Vehicle	CKD + 25(OH)D_3_ + 1,25(OH)_2_D_3_
	= 11	n = 8	n = 13	n = 10
Muscle pro-fibrotic factor Transforming growth factor-β1 (TGF-β1)	1.1 ± 1.0	1.1 ± 0.6	3.4 ± 0.4 ^A,C^	1.5 ± 0.4
Angiotensin (Agt)	1.1 ± 0.4	1.2 ± 0.9	5.1 ± 0.5 ^A,C^	2.3 ± 0.6 ^A^
Plasminogen activator inhibitor-1 (PAI-1)	1.1 ± 0.3	1.2 ± 0.8	3.2 ± 0.3 ^A,C^	1.9 ± 0.3 ^A^
Smad3	1.1 ± 0.3	1.3 ± 0.6	2.1 ± 0.3 ^A,C^	1.3 ± 0.2
Muscle anti-fibrotic factor BMP7	1.1 ± 0.7	1.4 ± 0.6	0.3 ± 0.2 ^B,C^	0.8 ± 0.2
IL-13Rα2	1.1 ± 0.3	1.3 ± 0.6	0.4 ± 0.2 ^B,C^	0.7 ± 0.2

Sham and CKD mice were treated with 25(OH)D_3_ and 1,25(OH)_2_D_3_ (75 μg/kg per day and 60 ng/kg per day, respectively) or ethylene glycol as vehicle for 6 weeks. Gastrocnemius muscle was used for mRNA expression study. Data are expressed and analyzed as in Table 1.

### Repletion of vitamin D ameliorates muscle fat infiltration in CKD mice

**Figure 5:**
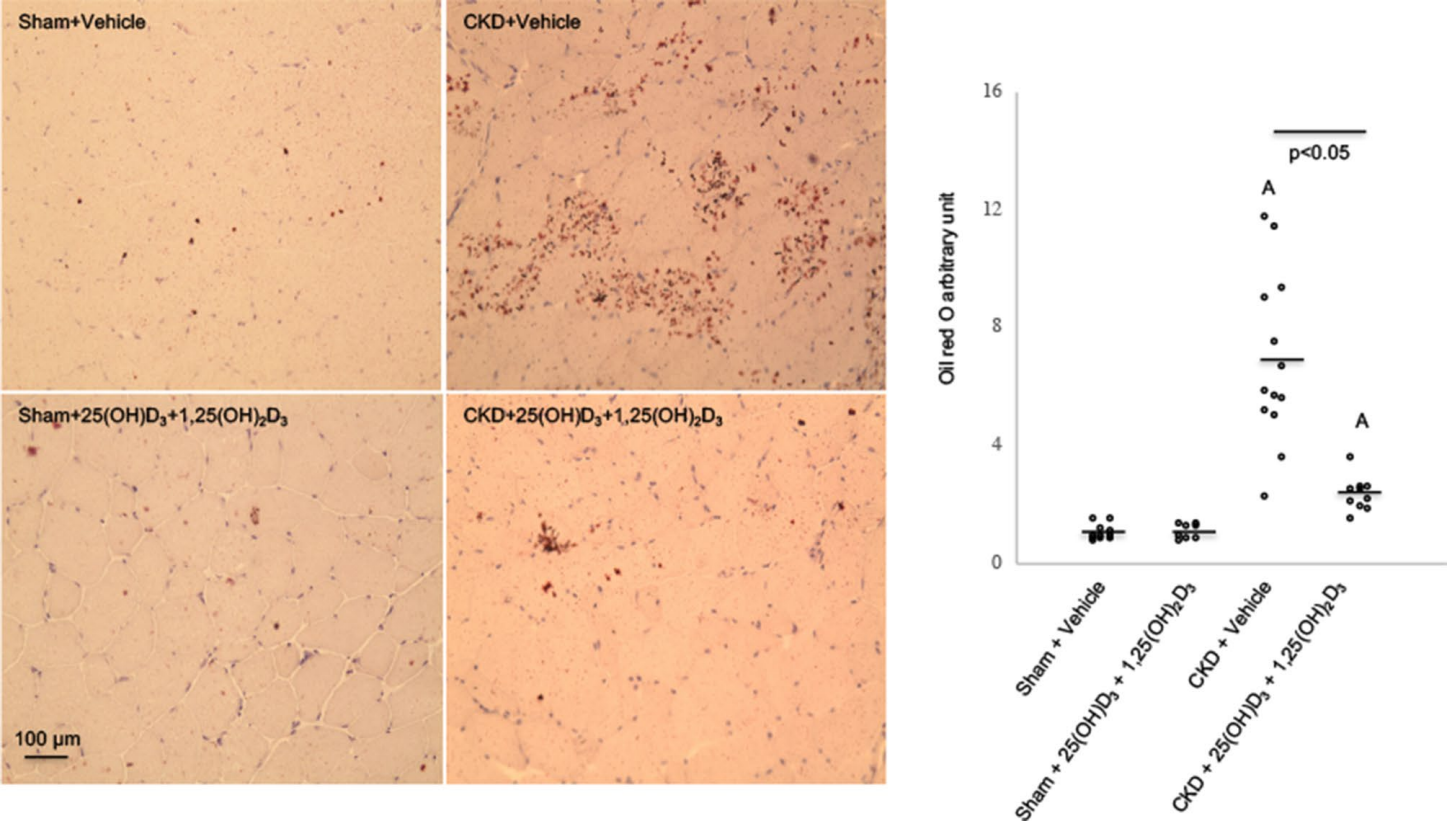
Vitamin D repletion attenuates muscle fat infiltration in CKD mice. Visualization and quantification of fatty infiltration by Oil Red O analysis in gastrocnemius muscle. Magnification is x20 for gastrocnemius muscle. Intensity for Oil Red O staining was recorded. Final results were expressed in arbitrary units, with one unit being the mean staining intensity in Sham + Vehicle mice. Difference among various groups of animals were analyzed as in Fig. 1.

We evaluated fat infiltration of the gastrocnemius muscle in mice. Representative results of Oil Red O staining of muscle section are shown (Fig. 5). We quantified intensity of muscle fat infiltration in mice and observed that vitamin D repletion attenuated muscle fat infiltration in CKD mice.

### Repletion of vitamin D modulates skeletal muscle mass regulation signaling in CKD mice

**Figure 6:**
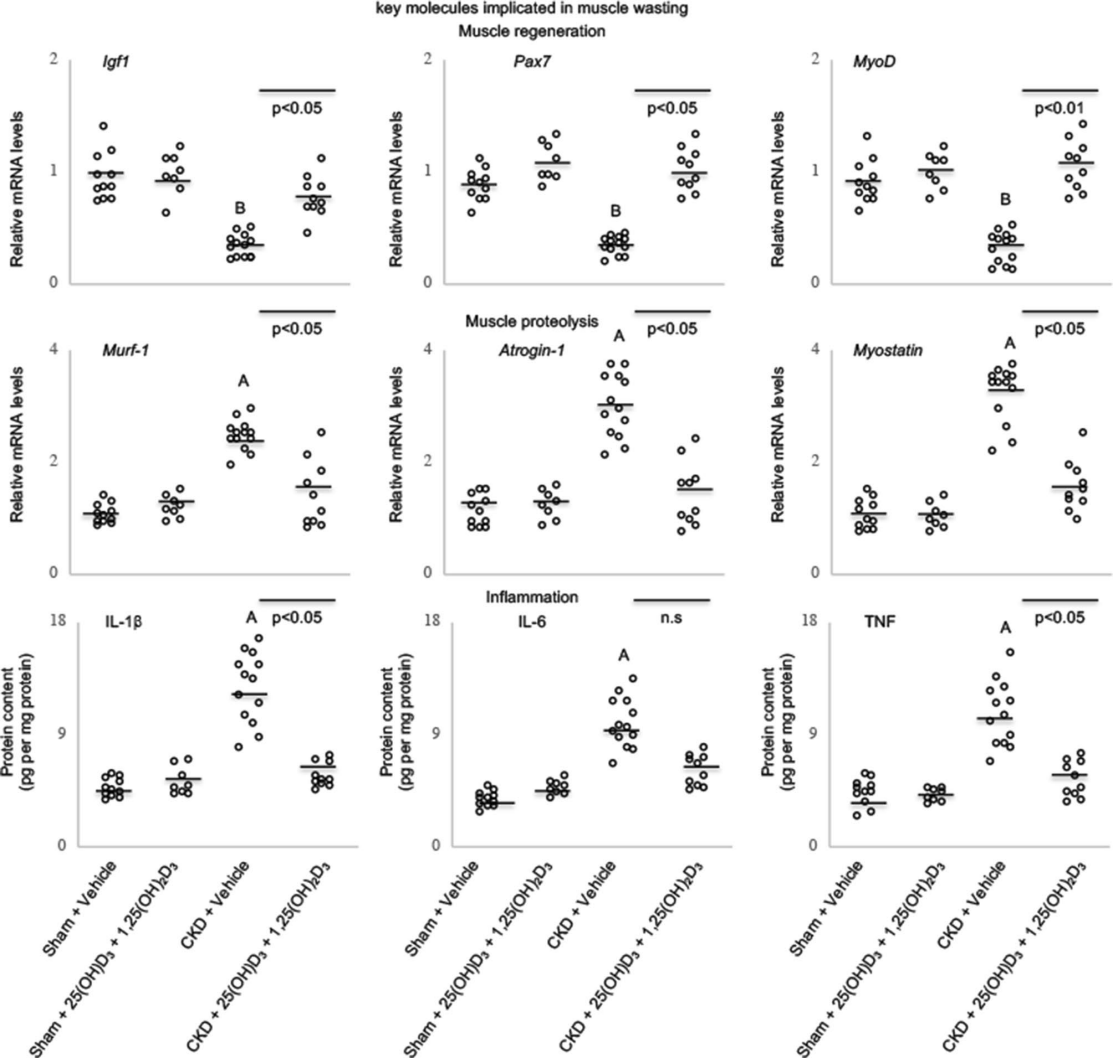
Vitamin D repletion modulates muscle mass signaling pathways in CKD mice. Gene expression of key molecules associated with muscle regeneration (IGF-1, Pax7 and MyoD), myogenesis (Murf-1, Atrogin-1, myostatin) and protein content of inflammatory cytokine (IL-1β, IL-6, and TNF) in gastrocnemius muscle were shown. Results are analyzed and expressed as in Fig. 1.

Vitamin D repletion improved muscle regeneration and myogenesis by increasing gastrocnemius muscle expression of pro-myogenic factors (IGF-1, Pax7 and MyoD) and decreasing expression of negative regulators of muscle mass (Murf-1, Atrogin-1 and myostatin) as well as decreasing muscle protein contents of inflammatory cytokines IL-1β, IL-6 and TNF (Fig. 6).

### Molecular mechanism by RNAseq analysis

**Figure 7:**
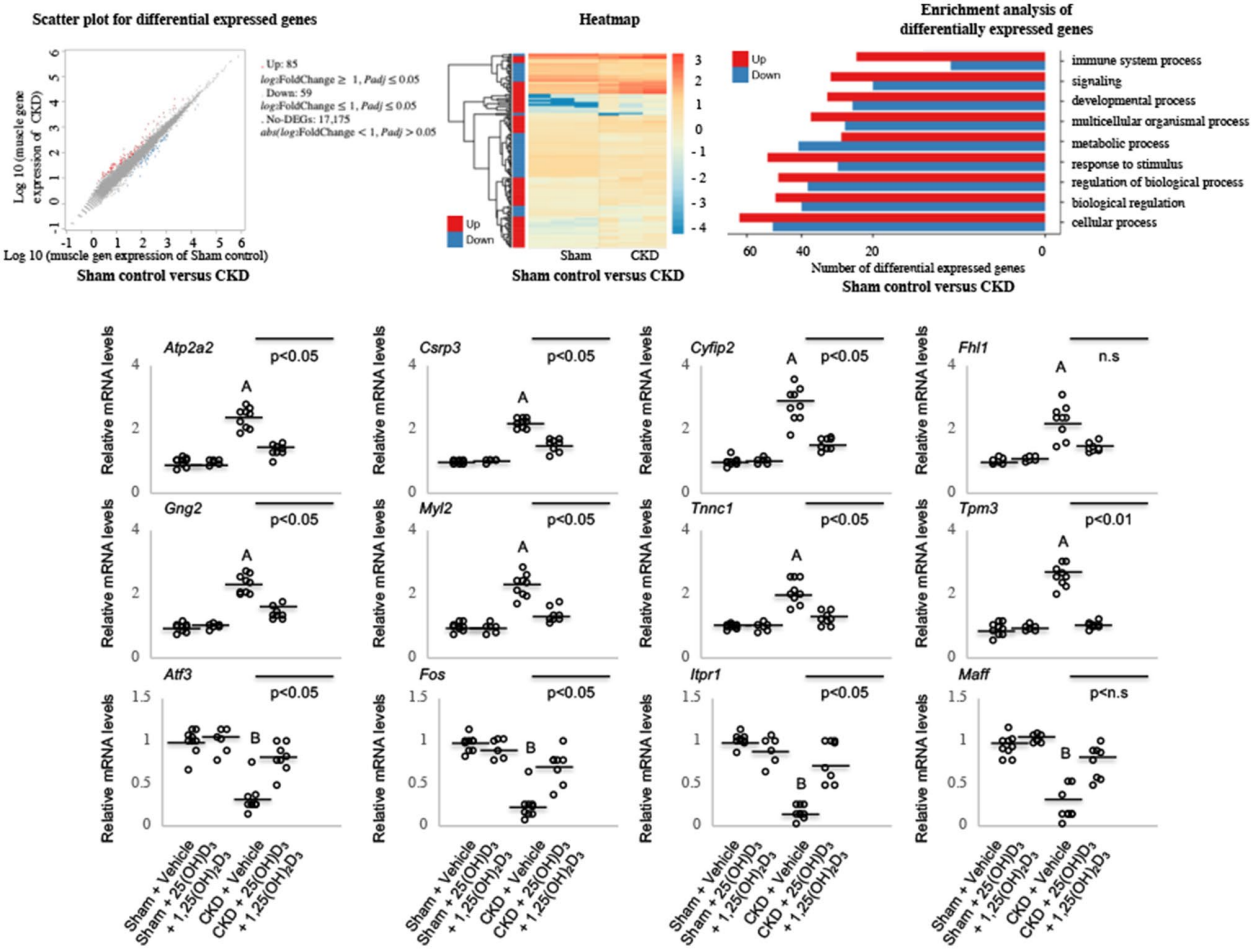
RNAseq analysis of gastrocnemius muscle. Summary of the number of differentially expressed genes (DEG) between CKD and sham control mice. We performed 5/6 nephrectomy and sham operation in 12-month old male C57BL/6J mice. Unbiased muscle RNAseq analyses were performed on the gastrocnemius muscle in 12-month old CKD mice versus age-appropriate control mice. Hierarchical clustering heatmap of DEG in CKD mice versus sham control mice. Gene ontology enrichment analysis for those DEG. To study the effects of vitamin D repletion in CKD mice, we further performed quantitative real-time PCR on gastrocnemius muscle from 4 groups of younger mice (3 months of age at sacrifice), *i.e.,* Sham + Vehicle, Sham + 25(OH)D_3_ + 1,25(OH)_2_D_3_, CKD + Vehicle as well as CKD + 25(OH)D_3_ + 1,25(OH)_2_D_3_ to determine the relative quantity of each targeted gene. Final results were expressed in arbitrary units, with one unit being the mean mRNA level in Sham + Vehicle mice. Results are analyzed as in Fig. 1.

**Figure 8:**
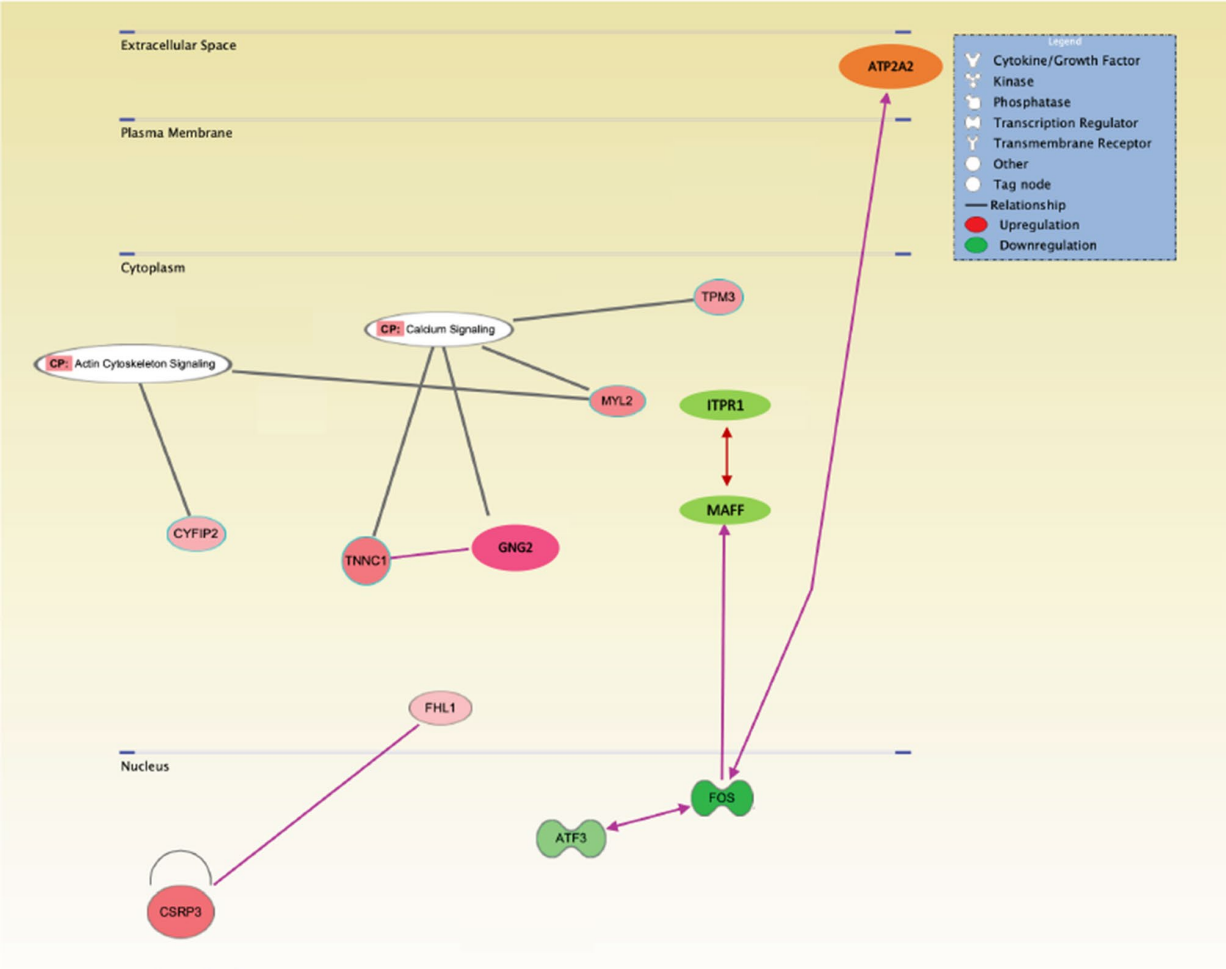
Functional annotation network. Ingenuity Pathway Analysis of alternations in energy metabolism, skeletal and muscular system development and function, and organismal injury and abnormalities in CKD mice relative to sham mice. Data were analyzed through the use of IPA (QIAGEN Inc., https://www.qiagenbioinformatics.com/products/ingenuity-pathway-analysis)^82^. The colored genes in the networks are differentially expressed between CKD mice relative to sham mice. Node color represents the expression status. Red: up-regulated in CKD mice relative to sham mice; green: down-regulated in CKD mice relative to sham mice. Increased expression of genes are ATP2A2, CSRP3, CYFIP2, FHL1, GNG2, MYL2, TNNC1 and TPM3, and decreased expression of genes are ATF3, FOS, ITPR1 and MAFF.

We studied differential expression of muscle mRNA between CKD and control mice in 12-month old animals using RNAseq analysis. We identified ~ 17,000 genes in both groups. We identified 85 upregulated genes and 59 downregulated genes in CKD mice versus sham mice (Fig. 7). Identities of these differentially expressed genes (DEG) are listed in Table 4. Hierarchical clustering heatmap showed the high degree of reproducibility in samples. Detailed GO pathway analysis revealed the top differentially expressed genes are involved in cellular processes, biological regulation, and metabolic processes in CKD mice versus sham mice. An Ingenuity Pathway Analysis enrichment test revealed important pathogenetic molecular pathways related to energy metabolism, skeletal and muscular system development and function, and organismal injury and abnormalities in CKD mice relative to control mice (Fig. 8). Upregulated genes were ATP2A2, CSRP3, CYFIP2, FHL1, GNG2, MYL2, TNNC1 and TPM3 whereas downregulated genes are ATF3, FOS, ITPR1 and MAFF in CKD mice versus sham mice. The functional significance of each of these DEG is listed (Table 3). Collectively, gene expression involved in muscle and neuron regeneration is impaired in CKD mice. In addition, expression of genes involved in enhanced muscle thermogenesis and fibrosis is increased in CKD mice. We then performed qPCR analysis in the muscle specimens in the experimental mice (3 months old) in the present study. Importantly, 25(OH)D3 and 1,25(OH)2D3 repletion normalized these top 12 DEG in CKD mice in the present study (Fig. 7).

**Table 4:** Muscle ingenuity analysis of canonical signaling *p* thways in wild type CKD mice versus wild type control mice.

Upregulated DEG	Functional significance and references
ATP2A2	Stimulates of UCP-1 independent beige thermogenesis^68^
CSRP3	Inhibition of myotube differentiation^70^
CYFIP2	Pro-apoptosis^75^
FHL1	Activates myostatin signaling and promotes atrophy in skeletal muscle^76^
GNG2	Adipocyte morphology and metabolic derangements^78^
MYL2	Associated with muscle fiber differentiation and fiber type transitions^71^
TNNC1	Biomarker for muscle depolarization^72^
TPM3	Promotes slow myofiber hypotrophy and associated with generalized muscle weakness^77^
Downregulated DEG	Functional significance
ATF3	Marker of neural injury, reduces the regeneration of neurons^74^
FOS	Associated with decreased skeletal muscle regeneration^73^
ITPR1	Impairment of muscle regeneration and mitochondrial dysfunction in myopathy patients^79^
MAFF	Involvement in brown adipose tissue thermogenesis^69^

We focus on pathways related to energy metabolism, skeletal and muscular system development and function, nervous system development and function as well as organismal injury and abnormalities. Functional significance of those differentially expressed genes are listed.

## Discussion

Our results showed that CKD mice were 25-hydroxyvitamin D3 and 1,25-dihydroxyvitamin D3 insufficient. Serum concentration of 25-hydroxyvitamin D3 status is influenced by the interplay between VDBP and free 25-hydroxyvitamin D3, which may be disrupted in the setting of CKD, perhaps from the urinary loss of VDBP. We measured serum concentrations of VDBP and 25-hydroxyvitamin D3 in mice. Interestingly, serum concentration of VDBP was elevated in CKD mice versus control mice (Table 1), implying that low free serum 25-hydroxyvitamin D3 concentrations rather than low serum VDBP is the cause of low serum total 25-hydroxyvitamin D3. This is distinct from the observation that low serum VDBP was more prevalent than low free serum concentration of 25-hydroxyvitamin D3 in CKD patients^23^. We then evaluated the effect of vitamin D repletion in CKD mice. Our results suggest that repletion of vitamin D attenuated adipose tissue browning and skeletal muscle wasting in CKD mice.

Hypermetabolism is a cardinal feature of cachexia in CKD^1,2^. Our results confirmed that hypermetabolism was present in CKD mice which was normalized by vitamin D repletion. Resting energy expenditure (REE) of CKD patients showed considerable variability, which may be influenced by degree of renal dysfunction, hyperparathyroidism, inflammation and the dialysis procedure^24–26^*.* Several investigations have reported that non-dialysis CKD patients have REE which may be similar or even slightly lower than that of age- and gender-matched healthy controls ^27,28^. These results may reflect the variable presence of cachexia in the pre-dialysis CKD population. Ikizler et al. carefully studied ESRD patients on maintenance hemodialysis and showed higher REE during resting conditions which was further increased during hemodialysis, compared with healthy controls^29^. Wang et al. studied a sizable population of 251 peritoneal dialysis patients, who were classified by REE tertiles. They showed that patients in the upper and middle tertiles of REE showed a significantly higher risk of all-cause mortality compared with those patients in the lower REE tertile^30^. Their findings highlight the significant negative prognostic effect of increased REE in CKD patients on long-term outcomes.

Adipose tissue and muscle are both involved in energy metabolism. Uncoupling protein (UCP)-1 is important for thermogenesis while UCP-2 and UCP-3 regulate mitochondrial energy metabolism^31^. Increased expression of adipose and muscle UCPs have been implicated in various diseased-associated cachexia^6,32,33^. UCPs are mitochondrial inner membrane proteins positioned in the same membrane as the ATPase, which is also a proton channel. UCPs and ATPase work in parallel with UCPs generating heat and ATP synthase generating ATP. Increased UCPs expression stimulates thermogenesis while inhibiting ATP synthesis^31^. Adipose tissue and muscle UCPs content was increased while adipose tissue and muscle ATP content was significantly decreased in CKD mice versus sham mice (Fig. 2). Vitamin D repletion attenuated perturbations of UCPs and ATP content in adipose tissue and muscle in CKD mice. Vitamin D insufficiency aggravates adipose tissue and muscle metabolism. 1,25-dihydroxyvitamin D3 suppresses expression of UCPs, cell differentiation as well as mitochondrial respiration in brown adipocytes^33,34^. Recent data also confirmed that vitamin D3 modulates UCP3 expression in muscle via the binding site consensus sequences of VDR on the UCP-3 promoter region^35^.

CKD mice exhibited increased expression of adipose tissue thermogenic genes (Pgc1*α*, Cidea, Prdm16 and Dio2) and elevated skeletal muscle gene expression involved in fatty acid oxidation (Ppar*α*, Ppar*δ* and Cpt1*α*) (Fig. 2). Fatty acids are essential elements of all cells and are important energy substrates. Increased muscle fatty acid metabolism stimulates energy expenditure and reduces mitochondrial energetics in humans^36^. Pgc1*α*, an important transcriptional coactivator, in cooperation with several other transcription factors, is involved in adipose tissue thermogenesis and muscle metabolism^37,38^. Increased expression of Pgc1*a* and its transcriptional co-activators promotes mitochondrial fatty acid oxidation and energy expenditure. In tissue where UCP-3 co-exists with UCP-2 (skeletal muscle and adipose tissue), UCP-3 and UCP-2 may act in concert in the overall regulation of fatty acid oxidation and energy expenditure^39^. Vitamin D repletion attenuated increased expression of both adipose tissue (Pgc1*α*, Cidrea, Prdm16 and Dio2) and skeletal muscle (Ppar*α*, Ppar*δ*, Cpt1*α*, Pgc1*α* and Pgc1*β*) thermogenic genes in CKD mice (Fig. 2).

We demonstrated the presence of BAT marker UCP-1 (Fig. 2) and the increased expression of beige adipocyte surface markers (CD137, Tmem26 and Tbx1) in inguinal WAT of CKD mice (Fig. 3). Repletion of vitamin D attenuated expression of these selective molecular markers of beige adipocytes in CKD mice. Cox2-associated prostaglandins act as paracrine signals and promote beige adipogenesis in inguinal WAT in mice^40,41^. We show that vitamin D repletion normalized over-expressed inguinal WAT Cox2 and Pgf2α mRNA level in CKD mice (Fig. 3). WAT secretes a plethora of inflammatory mediators in rodent models of cachexia. Vitamin D repletion attenuated over-expressed WAT protein content of IL-1β, IL-6 and TNF in CKD mice. Supplementation of 1,25-dihydroxyvitamin D3 decreases production of IL-1β, IL-6 and TNF in epididymal adipose tissue in mice and in human pre-adipocytes^22,42–44^. We profiled transcription factors involved in inflammatory pathways in CKD mice. Inguinal WAT of CKD mice exhibited increased expression and protein content of NFκB/TLR2 signaling pathways (NFκB p50/p65, Iκκ-α, Tlr2, MyD88 as well as Traf6). 1,25-dihydroxyvitamin D3 inhibits NFκB activation. Interaction of MyD88 and Traf6 is critical for Tlr2-mediated transactivation of NFκB and subsequent pro-inflammatory response^45^. We showed that repletion of vitamin D normalized inguinal WAT expression and protein content (Cox2 and Pgf2α; NF-κB p50 and p65, Tlr2, MyD88 and Traf6) and protein content of inflammatory cytokines (IL-1β, IL-6 and TNF) involved in promoting beige adipogenesis (Fig. 3). 1,25-dihydroxyvitamin D3 suppresses expression and protein content of Tlr2 in human cells^46^.

We studied the impact of vitamin D repletion on muscle fiber morphology and in vivo muscle function in CKD mice. To study muscle morphology, we chose to study two muscles known to have different fiber type compositions, namely soleus and tibialis anterior muscle. Soleus is a slow oxidative muscle while tibialis anterior is a fast glycolytic muscle^47^. Vitamin D repletion normalized average cross-sectional area of both the soleus and tibialis anterior muscles and attenuated in vivo muscle function (grip strength and rotarod activity) in CKD mice (Fig. 4). Our results are supported by previous reports that vitamin D significantly increased cross-sectional area of mouse C2C12 muscle cells^48^. Low vitamin D status is linked to suppressed skeletal muscle tropism and contraction^15^. Low serum vitamin D concentrations are associated with decreased skeletal muscle performance and an increase in the incidence of falls as well as risk of fractures^17,49,50^. In patients with diabetic nephropathy and CKD, treatment with paricalcitol was associated with no significant difference in glomerular filtration rate (GFR) measured directly with ^51^Cr-EDTA, but a significant decline in estimated GFR using an estimation formula employing serum creatinine. Serum creatinine increased significantly by a mean by 18 mmol/l despite no significant change in measured GFR, implying an increase in muscle mass in these patients^51^. 1,25-dihydroxyvitamin D3 increased myogenic gene expression and inhibited myostatin expression, a negative regulator of muscle mass in C2C12 cells^48,52^.

Muscle fat infiltration is a significant predictor of both muscle function and mobility function in older adults and across a wide variety of comorbid conditions such as diabetes, spinal cord injury and kidney disease, suggesting that increased muscle fat infiltration may at least partially account for a loss of strength and mobility seen in patients with CKD^53–55^. Muscle adipose tissue may release pro-inflammatory cytokines within the muscle and impair the local muscle environment, impair blood flow or increase the rate of lipolysis within skeletal muscle resulting in an increased concentration of glucose within the skeletal muscle itself followed by insulin resistance^56,57^. We evaluated the effects of vitamin D repletion on muscular fat infiltration content in CKD mice. Our results clearly demonstrated that vitamin D supplementation attenuated muscle fat infiltration in CKD mice (Fig. 5).

We investigated the impact of vitamin D repletion on signaling molecules that modulate muscle mass metabolism in CKD mice. Repletion of vitamin D attenuated the aberrant expression of genes involved in muscle regeneration (Igf1, Pax7, MyoD), proteolysis (Murf-1, Atrogin-1, myostatin) and inflammatory cytokines (IL-1*β*, IL-6, TNF) (Fig. 6). Myostatin impairs the growth of myocytes while stimulating the growth of muscle fibroblasts by involving the activation of several signaling pathways^58^. Inhibition of myostatin reverses muscle fibrosis through apoptosis^58^. Follistatin inhibits myostatin activity in vitro and stimulates muscle growth in vivo^59^. Vitamin D decreases muscle myostatin expression and increases muscle satellite cell regeneration^60^.

Chronic inflammation is an important factor for CKD-associated cachexia. TNF induces anorexia via peripheral and central nervous system mechanisms. Vitamin D supplementation reduced serum concentration of TNF in CKD patients^12,13^. Observational studies demonstrate that low serum vitamin D concentrations are correlated with increased concentrations of circulating inflammatory biomarkers such as C-reactive protein and IL-6^61^. Supplementation of vitamin D decreased serum concentrations of inflammatory biomarkers^13,61^. Vitamin D modulates immunity. Vitamin D dosage-dependently inhibited NF-*k*B activity and reduced IL-6 and TNF production in monocytes^62^. Moreover, vitamin D inhibited production of pro-inflammatory cytokines and proliferation of T lymphocytes^63^. T-cell cytokines modulate vitamin D metabolism in monocytes^64^.

Recent findings suggest that increased serum levels of PTH stimulates WAT browning and muscle wasting in mouse models of CKD and cancer^65^. CKD mice in this study had elevated circulating PTH levels. We found that the observed improvement in muscle wasting and adipose tissue browning was accompanied by normalization of 25-hydroxyvitamin D3 and 1,25-dihydroxyvitamin D3 and attenuation of the serum PTH concentrations in CKD mice (Table 1). Serum PTH concentrations were still significantly elevated even with successful vitamin D repletion. Based on these results, we postulate that the impact of secondary hyperparathyroidism may be less significant than vitamin D insufficiency in CKD-associated WAT browning and muscle wasting. To exclude the effects of PTH in CKD-associated cachexia in our experiment, we will need to perform parathyroidectomy in mice, but this is beyond the scope of the study.

We explored the mechanistic links between vitamin D repletion and the improvement of muscle morphology and muscle function in CKD mice. Skeletal muscle fibrosis is correlated with decreased muscle inflammation and muscle weakness in CKD patients^66^. Importantly, we showed that vitamin D repletion normalized soleus collagen content in CKD mice (Fig. 4). We measured gastrocnemius muscle expression of 84 key genes involved in tissue fibrosis in CKD versus control mice. We showed that vitamin D repletion exerts its antifibrotic effects on skeletal muscle by inhibiting the expression of pro-fibrotic genes (TGFβ1, Agt, PAI-1 and Smad3) while stimulating the expression of anti-fibrotic genes (BMP7 and IL13Rα2) in CKD mice (Table 3).

In addition to the known action of vitamin D metabolites on regulating mitochondrial function and muscle mass regulation, it is estimated that the vitamin D–endocrine system regulates approximately 3% of the human genome^67^. We therefore performed muscle RNAseq analysis in gastrocnemius muscle as an unbiased, systems biology approach to the molecular mechanism of vitamin D repletion in CKD mice. We demonstrated clear differentiation in muscle transcriptomics in CKD mice versus sham mice. Deficiency of vitamin D in CKD mice alters the muscle expression of genes involved in important biological processes as listed in Fig. 7. The functional significance of each of these DEG with relevant references were listed (Table 3 with reference 68 to 79). Subsequent IPA analysis revealed the important molecular mechanisms in vitamin D deficient CKD-associated muscle wasting. Downregulation of muscle expression of ITPR1, MAFF, ATF3 and FOS have been associated with upregulation of ATP2A2. Upregulated muscle ATP2A2 expression was associated with increased beige fat thermogenesis^68^. Increased muscle expression of FHL1 stimulates expression of CSRP3 and as a result, promotes muscle wasting^70,76^. Upregulation of CYFIP2 and MYL2 stimulates muscle apoptosis via actin cytoskeleton signaling^71,75^. In addition, increased muscle expression of TNNC1, GNG2 and TPM3 promotes myofiber atrophy and is correlated with muscle weakness via muscle calcium signaling^72,77,78^ (Fig. 8). Importantly, we show that vitamin D repletion normalized those top 12 differentially expressed genes in CKD mice (Fig. 7).

In conclusion, our results showed that CKD mice were vitamin D insufficient. Repletion of vitamin D normalized serum vitamin D concentrations in CKD mice. Most importantly, repletion of vitamin D ameliorated white adipose tissue browning and muscle wasting in CKD mice (Supplemental Fig. 3S). Supplementation of vitamin D may present a mechanistically sound and inexpensive therapeutic strategy as an anti-WAT browning and anti-muscle wasting agent for patients with CKD.

## Materials and methods

### Experimental design

The study was in compliance with the approved animal protocol by the Institutional Animal Care and Use Committee (IACUC) at the University of California, San Diego. Schematic study design is listed (Supplemental Fig. 2S). Male C57BL/6 J mice at 6 weeks of age were used for the study. We surgically induced CKD in mice by performing 5/6 nephrectomy as well as performing the sham operation in control mice^6^. CKD and sham mice were treated with 25(OH)D_3_ (Sigma, Catalog 739,650-1ML, 75 μg/kg/day), 1,25(OH)_2_D_3_ (Sigma, Catalog 740,578-1ML, 60 ng/kg/day) or vehicle control (ethylene glycol) using subcutaneous osmotic Alzet 2006 pump for 6 weeks. Oxygen consumption (VO_2_) was measured by using Oxymax indirect calorimetry (Columbus Instrument) and lean mass and fat content of mice were analyzed by quantitative magnetic resonance method (EchoMRI-100™, Echo Medical System)^6^. Muscle function (grip strength and rotarod activity) in mice was assessed using grip strength meter (Model 47,106, UGO Basile) and rotarod performance test (model RRF/SP, Accuscan Instrument), respectively^6^.

### Serum and blood chemistry

Serum concentration of bicarbonate, Ca and Pi was assessed. Concentrations of BUN, serum creatinine, 25(OH)D_3_, 1,25(OH)_2_D_3_, parathyroid hormone (PTH) and vitamin D binding protein (VDBP) were analyzed (Supplemental Table 4S).

### Tissue adenosine triphosphate and protein levels

Adenosine triphosphate (ATP) and uncoupling protein (UCP) contents in tissue homogenates were measured. Protein contents of pro-inflammatory cytokines (IL-1β, IL-6 and TNF) in adipose and muscle tissue lysate were quantified (Supplemental Table 4S).

### Muscle fiber size and muscle collagen content

Muscle fiber cross-sectional area of soleus and tibias anterior was measured^6^. Excised soleus muscles were hydrolyzed and hydroxyproline content was calculated (Supplemental Table 4S).

### Quantification of fatty infiltration in skeletal muscle

Fresh gastrocnemius muscles were preserved in isopentane/liquid nitrogen. Dissected muscle samples were incubated with Oil Red O (Oil Red O Solution, catalog number O1391-250 ml, Sigma Aldrich). Detailed procedures for Oil Red O staining were in accordance with published protocol^80^. We followed a recently established protocol to quantify muscle fat infiltration. Acquisition and quantification of images were analyzed using ImageJ software (https://rsbweb.nih.gob/ij/)^81^.

### RT^2^ Profiler PCR array for muscle fibrosis

Muscle fibrotic and anti-fibrotic gene expression was analyzed (Qiagen, Catalog 330,231 PAMM-120ZA). Detailed information for a total of 84 genes is listed in Supplemental Table 2S.

### Muscle RNAseq analysis

We performed 5/6 nephrectomy and sham operation in 12-month old male C57BL/6 J mice. Total gastrocnemius muscle RNA was isolated in CKD and sham mice (3 mice in each group) using Trizol (Life Technology) followed by RNeasy mini kit (Qiagen) for further purification. The extracted muscle RNA samples were analyzed using Agilent 2100 Bioanalyzer (Agilent RNA 6000 Nano Kit). Samples were used to construct cDNA libraries (Illumina) and sequenced through an Illumina HiSeq2000 platform at BGI Hong Kong (www.bgi.com). The raw RNAseq data were filtered into clean reads, followed by mapping to the mouse reference genome using HISAT. The gene expression level for each sample was analyzed using RSEM quantification tool. Based on the gene expression level, differentially expressed genes (DEG) between CKD and sham mice were identified using DESeq2 algorithms. Biological function analysis of the DEG was enriched by Gene Ontology (GO) and Kyoto Encyclopedia of Genes and Genomes (KEEG) pathway. To identify pathways related to phenotypic differences of the muscle between CKD and sham mice, DEG between CKD and sham mice were analyzed through the use of IPA (QIAGEN Inc., https://www.qiagenbio-informatics.com/products/ingenuity-pathway-analysis)^82^.

### Gene expression analysis

Total RNA from adipose and gastrocnemius muscle samples were isolated using TriZol (Life Technology) and reverse-transcribed with SuperScript III Reverse Transcriptase (Invitrogen). Quantitative real-time RT-PCR of target genes were performed using KAPA SYBR FAST qPCR kit (KAPA Biosystems). Expression levels were calculated according to the relative 2^-*DD*Ct^ method^6^. All primers are listed in Supplemental Table 5S.

### Statistical analysis

Continuous variables are expressed as mean ± S.E.M. We assessed the statistical significance of differences between groups using two-sample t-tests. All tests were two-sided. A *p* value less than 0.05 was considered significant. Statistical analyses were performed using SPSS software version 16.0 for Macintosh.

## Supplementary information

Supplementary information
