# Supplementary material for: Vitamin D ameliorates adipose browning in chronic kidney disease cachexia
Source: Sci Rep. 2020 Aug 25;10:14175. doi: 10.1038/s41598-020-70190-z (PMC7447759; doi:10.1038/s41598-020-70190-z)
Supplement: Supplementary file 1 — Supplementary information [file 41598_2020_70190_MOESM1_ESM.pdf]

Vitamin D ameliorates adipose browning in chronic kidney disease cachexia

Wai W Cheung[1*], Wei Ding[2*], Hal M Hoffman[3], Zhen Wang[4], Sheng Hao[5], Ronghao Zheng[6], Alex Gonzalez[1], Jian-Ying Zhan[7], Ping Zhou[8], Shiping Li[9], Mary C Esparza[10], Richard L Lieber[11], and Robert H Mak[1]

[1] Pediatric Nephrology, Rady Children's Hospital San Diego, University of California, San Diego
[2] Division of Nephrology, Shanghai Ninth People's Hospital, School of Medicine, Shanghai Jiaotong University, Shanghai, China
[3] Department of Pediatrics, University of California, San Diego
[4]Department of Pediatrics, Shanghai General Hospital, Shanghai Jiao Tong University, Shanghai, China
[5]Department of Nephrology and Rheumatology, Shanghai Children's Hospital, Shanghai Jiao Tong University, Shanghai, China
[6]Department of Pediatrics, Hubei Maternal and Child Health Hospital, Wuhan, China
[7] Children's Hospital, Zhejiang University, Hangzhou, China
[8] Department of Pediatrics, the 2nd Hospital of Harbin Medical University, Harbin, China
[9] College of Bioscience and Biotechnology, Yangzhou University, Yangzhou, China
[10] Department of Orthopedic Surgery, University of California, San Diego
[11] Rehabilitation Institute of Chicago, Chicago

* These authors contributed equally to this work.

Correspondence:
Robert H Mak
Division of Pediatric Nephrology
Department of Pediatrics
University of California, San Diego
9500 Gilman Drive, MC0630, La Jolla, California 92093-0630
P: 858-822-6717
F: 858-822-6776
E-mail: romak@ucsd.edu

Running headline: Vitamin D repletion attenuates uremic cachexia

Keywords: chronic kidney disease, vitamin D deficiency, cachexia, adipose tissue browning, muscle wasting

Supplemental figure legends

Figure 1S: **Food intake and weight gain in CKD mice**. Sham and CKD mice were treated with $25(OH)D_3$ and $1,25(OH)_2D_3$ (75 µg/kg per day and 60 ng/kg per day, respectively) or ethylene glycol as vehicle for 6 weeks. Four groups of mice were included: Sham + Vehicle (n=8), Sham + $25(OH)D_3$ + $1,25(OH)_2D_3$ (n=8), CKD + Vehicle (n=8) and CKD + $25(OH)D_3$ + $1,25(OH)_2D_3$ (n=8). Mice were fed *ad libitum* and weight gain was recorded. Data are expressed as mean ± SEM. CKD + Vehicle and CKD + $25(OH)D_3$ + $1,25(OH)_2D_3$ were compared to Sham + Vehicle and Sham + $25(OH)D_3$ + $1,25(OH)_2D_3$, respectively. ^{B} $p < 0.05$, significantly lower in CKD + Vehicle mice versus Sham + Vehicle mice. Results of CKD + Vehicle mice were also compared to CKD + $25(OH)D_3$ + $1,25(OH)_2D_3$ mice.

Figure 2S: **Schematic representation of the study design.**

Figure 3S: **Molecular mechanisms of muscle wasting in vitamin D repleted CKD mice.** Supplementation of $25(OH)D_3$ and $1,25(OH)_2D_3$ attenuates cachexia, improves energy homeostasis and attenuates adipose tissue browning and muscle wasting in CKD mice.

Supplemental Figure 1S

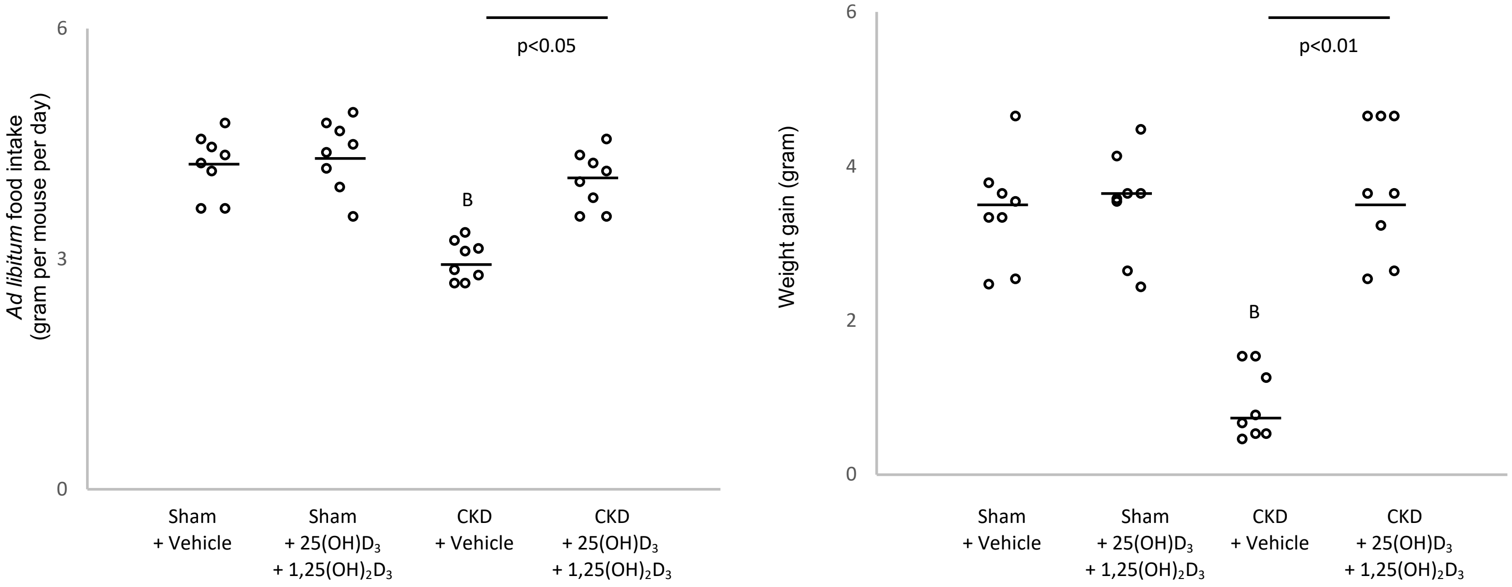

Supplemental Figure 2S

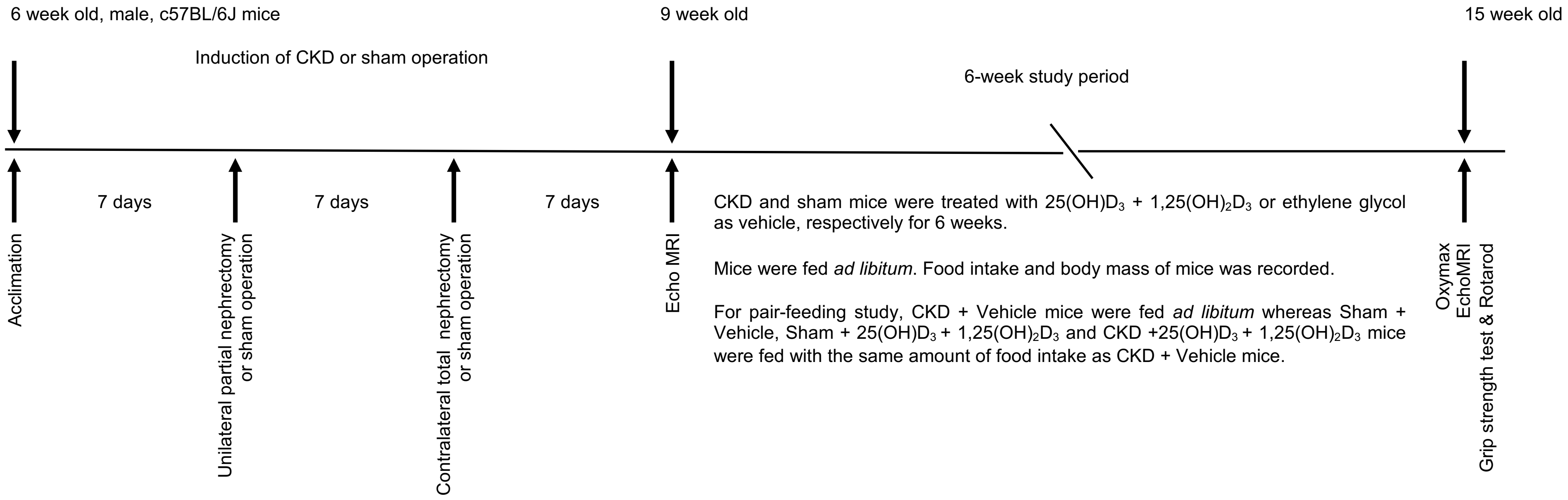

Supplemental Figure 3S

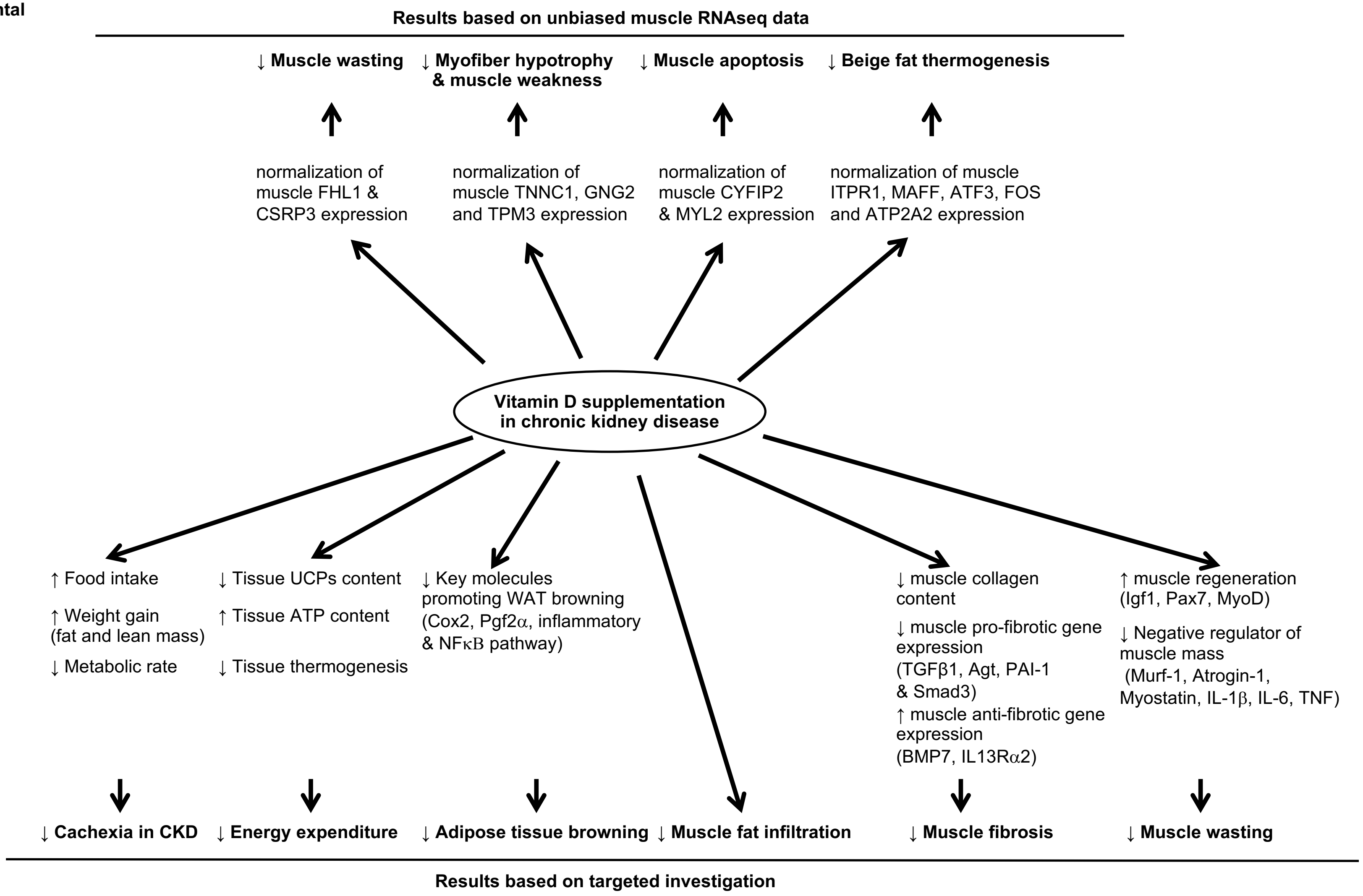

Supplemental Table 1S: Serum and blood chemistry of mice. Sham and CKD mice were treated with 25(OH)D_3 and 1,25(OH)$_2D_3$ (75 μg/kg per day and 60 ng/kg per day, respectively) or ethylene glycol as vehicle for 6 weeks. Data are expressed as mean ± SEM. ^{A} $p < 0.05$, significantly higher in CKD + Vehicle and CKD + 25(OH)D_3 + 1,25(OH)$_2D_3$ mice versus Sham + Vehicle and Sham + 25(OH)D_3 + 1,25(OH)$_2D_3$ mice, respectively. ^{B} $p < 0.05$, significantly lower in CKD + Vehicle and CKD + 25(OH)D_3 + 1,25(OH)$_2D_3$ mice versus Sham + Vehicle and Sham + 25(OH)D_3 + 1,25(OH)$_2D_3$ mice, respectively. ^{C} $p < 0.05$, significantly different between CKD + Vehicle and CKD + 25(OH)D_3 + 1,25(OH)$_2D_3$ mice.

	Sham +Vehicle (n=8)	Sham +25(OH)D_3 +1,25(OH)$_2D_3$ (n=8)	CKD +Vehicle (n=8)	CKD +25(OH)D_3 +1,25(OH)$_2D_3$ (n=8)
BUN (mg/dl)	27.5 ± 6.4	26.7 ± 7.4	65.7 ± 11.5^A	77.3 ± 22.0^A
Creatinine (mg/dl)	< 0.2	< 0.2	0.8 ± 0.5^A	0.6 ± 0.5^A
Bicarbonate (mmol/l)	27.6 ± 4.2	26.5 ± 6.2	25.9 ± 8.1	27.6 ± 6.6
25(OH)D_3 (ng/ml)	109.1 ± 19.4	120.1 ± 36.6	50.6 ± $21.6^{B,C}$	105.4 ± 24.3
1,25(OH)$_2D_3$ (pg/ml)	267.7 ± 50.8	265.3 ± 23.4	105.6 ± $52.4^{B,C}$	210.4 ± 62.8

Supplemental Table 2S: Genes assessed by RT^2 Profile PCR Array Mouse Fibrosis (PAMM-120ZA). The mouse Fibrosis RT^2 Profiler^{TM} PCR Array profiles the expression of 84 key genes involved in dysregulated tissue remodeling during the repair and healing of wounds. Specifically, this array contains genes encoding pro-fibrotic, anti-fibrotic, important molecules for extracellular matrix metabolism and cell adhesion, inflammatory cytokines and chemokines, growth factors, TGFβ signaling transduction molecules, molecules involved in epithelial-to-mesenchymal transition as well as additional genes important for fibrosis. The list of the 84 genes is shown as following.

Pro-Fibrotic: Acta2 (α-SMA), Agt, Ccl11 (Eotaxin), Ccl12, Ccl3 (MIP-1α), Ctgf, Grem1, Il13, Il13rα2, Snai1(Snail)

Anti-Fibrotic: Bmp7, Hgf, Ifng, Il10, Il13rα2

Extracellular Matrix & Cell Adhesion:
ECM Components: COL1A2, COL3A1
Remodeling Enzymes: Lox, Mmp1a (Collagenase 1), Mmp13, Mmp14, Mmp2 (Gelatinase A), Mmp3, Mmp8, Mmp9 (Gelatinase B), Plat (tPA), Plau (uPA), Plg, Serpina1a (a1-antitrypsin), Serpine1 (PAI-1), Serpinh1, Timp1, Timp2, Timp3, Timp4
Cellular Adhesion: Itga1, Itga2, Itga3, Itgav, Itgb1, Itgb3, Itgb5, Itgb6, Itgb8

Inflammatory Cytokines & Chemokines: Ccl11 (Eotaxin), Ccl12, Ccl3 (MIP-1a), Ccr2, Cxcr4, Ifng, Il10, Il13, Il13rα2, Il1α, Il1β, Tnf

Growth Factors: Agt, Ctgf, Edn1, Egf, Hgf, Pdgfα, Pdgfβ, Vegfα

Signal Transduction:
TGFβ Superfamily: Bmp7, Cav1, Dcn, Eng (EVI-1), Grem1, Inhbe, Ltbp1, Smad2, Smad3, Smad4, Smad6, Smad7, Tgfβ1, Tgfβ2, Tgfβ3, Tgfβr1 (ALK5), Tgfβr2, Tgif1, Thbs1, Thbs2
Transcription Factors: Cebpb, Jun, Myc, Nfkb1, Sp1, Stat1, Stat6

Epithelial-to-Mesenchymal Transition: Akt1, Bmp7, Col1α2, Col3α1, Itgαv, Itgβ1, Mmp2 (Gelatinase A), Mmp3, Mmp9, Serpine1 (PAI-1), Smad2, Snai1 (Snail), Tgfβ1, Tgfβ2, Tgfβ3, Timp1

Others: Bcl2, Fasl (TNFSF6)

Supplemental Table 3S: List of differential expressed genes in gastrocnemius muscle from CKD versus Sham mice

Gene ID	Expression (Sham)	Expression (CKD)	log2 Fold Change (CKD vs Sham)	P - adjusted	Up/Down-Regulation	P value	Symbol	Desciption
21952	180.48	1300.48	2.85	1.35907E-06	Up	1.80488E-09	Tnni1	troponin I, skeletal, slow 1
791403	19.30	138.73	2.85	2.76105E-09	Up	1.59423E-12	D830015G02Rik	RIKEN cDNA D830015G02 gene
140781	2091.69	12648.18	2.60	7.43233E-06	Up	1.41617E-08	Myh7	myosin, heavy polypeptide 7, cardiac muscle, beta
208154	2.83	17.01	2.59	0.001810488	Up	9.82654E-06	Btla	B and T lymphocyte associated
13009	138.21	799.16	2.53	8.57301E-06	Up	1.83152E-08	Csrp3	cysteine and glycine-rich protein 3
21924	296.21	1665.21	2.49	5.66203E-05	Up	1.43847E-07	Tnnc1	troponin C, cardiac/slow skeletal
20307	6.29	34.65	2.46	0.000328944	Up	1.15859E-06	Ccl8	chemokine (C-C motif) ligand 8
16819	7.19	38.27	2.41	0.000798278	Up	3.50304E-06	Lcn2	lipocalin 2
105005	2.86	15.05	2.40	0.007088458	Up	5.06772E-05	Fam84a	family with sequence similarity 84, member A
55985	7.13	37.15	2.38	7.10016E-05	Up	1.89252E-07	Cxcl13	chemokine (C-X-C motif) ligand 13
21955	493.73	2556.47	2.37	0.000349473	Up	1.29143E-06	Tnnt1	troponin T1, skeletal, slow
20201	6.36	32.77	2.36	0.007067429	Up	4.9785E-05	S100a8	S100 calcium binding protein A8 (calgranulin A)
12654	2.85	14.31	2.33	0.010002752	Up	8.08583E-05	Chi3l1	chitinase 3-like 1
15002	6.11	29.13	2.25	0.00576479	Up	3.96103E-05	H2-Ob	histocompatibility 2, O region beta locus
12478	5.39	25.57	2.25	0.012668527	Up	0.000108429	Cd19	CD19 antigen
17906	754.79	3554.30	2.24	0.003155739	Up	1.9679E-05	Myl2	myosin, light polypeptide 2, regulatory, cardiac, slow
98752	2.76	12.85	2.22	0.018510848	Up	0.000171011	Fcrla	Fc receptor-like A
23833	14.67	67.67	2.21	0.002354991	Up	1.33303E-05	Cd52	CD52 antigen
19354	19.31	86.56	2.16	0.000141437	Up	4.32829E-07	Rac2	RAS-related C3 botulinum substrate 2
15985	7.89	34.81	2.14	0.020770856	Up	0.000203883	Cd79b	CD79B antigen
268857	7.96	34.24	2.10	0.00351448	Up	2.23219E-05	Nlrc3	NLR family, CARD domain containing 3
232975	5.90	25.10	2.09	0.007298959	Up	5.39446E-05	Atp1a3	ATPase, Na+/K+ transporting, alpha 3 polypeptide
100041546	1.94	8.15	2.07	0.034917815	Up	0.000445571	Ly6c2	lymphocyte antigen 6 complex, locus C2
16818	4.42	18.38	2.06	0.037826426	Up	0.000492352	Lck	lymphocyte protein tyrosine kinase
70450	2.61	10.74	2.04	0.031234172	Up	0.000386128	Unc13d	unc-13 homolog D (C. elegans)
16994	6.38	25.90	2.02	0.040580969	Up	0.000569258	Ltb	lymphotoxin B
18985	3.54	14.37	2.02	0.040916563	Up	0.000581181	Pou2af1	POU domain, class 2, associating factor 1
20343	7.97	32.16	2.01	0.023793627	Up	0.000254161	Sell	selectin, lymphocyte
24108	8.87	35.71	2.01	0.040580969	Up	0.000557772	Ubd	ubiquitin D
272382	5.87	23.14	1.98	0.049659711	Up	0.000782595	Spib	Spi-B transcription factor (Spi-1/PU.1 related)
12525	3.80	14.77	1.96	0.049659711	Up	0.00079139	Cd8a	CD8 antigen, alpha chain
17392	13.67	53.05	1.96	6.31394E-05	Up	1.64055E-07	Mmp3	matrix metallopeptidase 3
18772	3.00	11.36	1.92	0.049631188	Up	0.000773741	Pkp1	plakophilin 1
29813	106.79	400.47	1.91	2.39497E-05	Up	5.53143E-08	Zfp385a	zinc finger protein 385A
19419	4.47	16.76	1.91	0.044638811	Up	0.000662243	Rasgrp1	RAS guanyl releasing protein 1
21973	6.55	24.41	1.90	0.049659711	Up	0.000780735	Top2a	topoisomerase (DNA) II alpha
18140	4.92	17.92	1.87	0.038750202	Up	0.000513855	Uhrf1	ubiquitin-like, containing PHD and RING finger domains, 1
17883	21.45	77.84	1.86	0.015675791	Up	0.000138484	Myh3	myosin, heavy polypeptide 3, skeletal muscle, embryonic
16069	7.45	26.87	1.85	0.049446039	Up	0.000765145	Igj	immunoglobulin joining chain
104886	16.33	57.31	1.81	0.000532623	Up	2.15276E-06	Rab15	RAB15, member RAS oncogene family
11938	2002.49	6990.06	1.80	0.010193759	Up	8.29909E-05	Atp2a2	ATPase, Ca++ transporting, cardiac muscle, slow twitch 2
215243	6.14	21.30	1.79	0.030494199	Up	0.000373276	Traf3ip3	TRAF3 interacting protein 3
14421	16.32	55.35	1.76	0.030086479	Up	0.000359599	B4galnt1	beta-1,4-N-acetyl-galactosaminyl transferase 1
54352	14.41	48.54	1.75	0.038750202	Up	0.000514611	Irx5	Iroquois related homeobox 5 (Drosophila)

20856	16.63	56.01	1.75	0.001104744	Up	5.2944E-06	Stc2	stanniocalcin 2
59006	423.83	1424.11	1.75	0.023015788	Up	0.000239541	Myoz2	myozenin 2
18784	28.19	90.29	1.68	0.01636965	Up	0.000147449	Pla2g5	phospholipase A2, group V
319616	5.74	18.14	1.66	0.04475211	Up	0.000668881	5930412G12Rik	RIKEN cDNA 5930412G12 gene
16997	9.64	30.26	1.65	0.046816238	Up	0.000708231	Ltbp2	latent transforming growth factor beta binding protein 2
76884	60.37	186.58	1.63	0.000529915	Up	2.05002E-06	Cyfip2	cytoplasmic FMR1 interacting protein 2
59069	803.75	2438.19	1.60	0.03295603	Up	0.000418634	Tpm3	tropomyosin 3, gamma
16364	15.16	45.69	1.59	0.048707791	Up	0.000749219	Irf4	interferon regulatory factor 4
12721	37.00	110.46	1.58	0.030392319	Up	0.000370274	Coro1a	coronin, actin binding protein 1A
15170	19.66	58.32	1.57	0.030392319	Up	0.0003686	Ptpn6	protein tyrosine phosphatase, non-receptor type 6
19228	13.15	37.61	1.52	0.04113497	Up	0.000586658	Pth1r	parathyroid hormone 1 receptor
16854	50.06	140.26	1.49	0.001671884	Up	8.97772E-06	Lgals3	lectin, galactose binding, soluble 3
20750	15.37	42.58	1.47	0.029123847	Up	0.000343049	Spp1	secreted phosphoprotein 1
14199	4470.29	12325.54	1.46	3.87222E-06	Up	6.48388E-09	Fhl1	four and a half LIM domains 1
14955	1870.49	5147.75	1.46	0.048707791	Up	0.000741809	H19	H19 fetal liver mRNA
544963	21.53	59.01	1.45	0.048707791	Up	0.000750908	Iqgap2	IQ motif containing GTPase activating protein 2
17060	15.09	40.72	1.43	0.044638811	Up	0.000658563	Blnk	B cell linker
20672	61.51	162.88	1.40	1.31149E-05	Up	2.87756E-08	Sox18	SRY-box containing gene 18
70719	37.19	98.31	1.40	0.020501909	Up	0.000196966	Hmha1	histocompatibility (minor) HA-1
26557	206.03	534.69	1.38	2.78158E-07	Up	2.55412E-10	Homer2	homer homolog 2 (Drosophila)
72690	14.70	38.07	1.37	0.040580969	Up	0.000569385	Grrp1	glycine/arginine rich protein 1
13717	96.65	249.39	1.37	2.63469E-06	Up	3.99292E-09	Eln	elastin
16205	15.54	39.64	1.35	0.026270663	Up	0.000294273	Gimap1	GTPase, IMAP family member 1
74100	36.83	93.52	1.34	0.023647884	Up	0.000251239	Arpp21	cyclic AMP-regulated phosphoprotein, 21
11829	208.37	528.39	1.34	4.76213E-08	Up	3.29959E-11	Aqp4	aquaporin 4
50706	104.46	264.70	1.34	7.00705E-10	Up	3.64129E-13	Postn	periostin, osteoblast specific factor
14702	50.63	124.98	1.30	0.014843507	Up	0.000129968	Gng2	guanine nucleotide binding protein (G protein), gamma 2
23893	71.04	166.64	1.23	0.039800265	Up	0.000542344	Grem2	gremlin 2 homolog, cysteine knot superfamily (Xenopus laevis)
11622	22.29	50.30	1.17	0.041250528	Up	0.000590688	Ahr	aryl-hydrocarbon receptor
245683	79.91	179.01	1.16	0.021321915	Up	0.000211754	Klhl34	kelch-like 34
16170	30.53	67.79	1.15	0.023013397	Up	0.000236525	Il16	interleukin 16
104099	62.33	138.17	1.15	0.022870304	Up	0.000233734	Itga9	integrin alpha 9
12984	33.14	71.68	1.11	0.021540668	Up	0.00021517	Csf2rb2	colony stimulating factor 2 receptor, beta 2, low-affinity (granulocyte-macrophage)
244418	43.80	94.21	1.11	0.009995831	Up	8.00776E-05	D8Ertd82e	DNA segment, Chr 8, ERATO Doi 82, expressed
22228	135.17	289.80	1.10	8.24964E-06	Up	1.71481E-08	Ucp2	uncoupling protein 2 (mitochondrial, proton carrier)
17110	41.04	86.24	1.07	0.04475211	Up	0.000669253	Lyz1	lysozyme 1
16848	34.72	71.55	1.04	0.030392319	Up	0.000370262	Lfng	LFNG O-fucosylpeptide 3-beta-N-acetylglucosaminyltransferase
23796	40.72	83.08	1.03	0.040580969	Up	0.000556477	Aplnr	apelin receptor
13358	195.24	396.30	1.02	0.037247395	Up	0.000479599	Slc25a1	solute carrier family 25 (mitochondrial carrier, citrate transporter), member 1
23956	108.51	218.15	1.01	0.026871493	Up	0.000305658	Neu2	neuraminidase 2
17472	44.72	89.67	1.00	0.027399337	Up	0.000313244	Gbp4	guanylate binding protein 4

Gene ID	Expression (Sham)	Expression (CKD)	log2 Fold Change (CKD vs Sham)	P - adjusted	Up/Down-Regulation	P value	Symbol	Desciption
20878	122.25	60.93	-1.00	0.002891001	Down	1.78611E-05	Aurka	aurora kinase A
55942	131.22	65.20	-1.01	0.00257903	Down	1.5487E-05	Sertad1	SERTA domain containing 1
11504	743.51	369.27	-1.01	0.001126253	Down	5.52754E-06	Adamts1	a disintegrin-like and metallopeptidase (reprolysin type) with thrombospondin type 1 motif, 1
16598	1013.68	501.71	-1.01	1.03554E-07	Down	7.77298E-11	Klf2	Kruppel-like factor 2 (lung)
56222	280.23	138.57	-1.02	0.022159085	Down	0.000222627	Cited4	Cbp/p300-interacting transactivator, with Glu/Asp-rich carboxy-terminal domain, 4
193740	380.13	187.74	-1.02	0.039342059	Down	0.000527813	Hspa1a	heat shock protein 1A
74155	1031.63	505.58	-1.03	5.76392E-07	Down	6.52714E-10	Errfi1	ERBB receptor feedback inhibitor 1
76933	609.09	298.18	-1.03	0.031320855	Down	0.000390629	Ifi27l2a	interferon, alpha-inducible protein 27 like 2A
15511	269.41	129.37	-1.06	0.011221206	Down	9.32995E-05	Hspa1b	heat shock protein 1B
83397	584.86	278.82	-1.07	0.000169723	Down	5.29191E-07	Akap12	A kinase (PRKA) anchor protein (gravin) 12
16438	604.17	286.70	-1.08	3.83925E-10	Down	1.77343E-13	Itpr1	inositol 1,4,5-trisphosphate receptor 1
16600	859.03	407.49	-1.08	2.04593E-05	Down	4.60715E-08	Klf4	Kruppel-like factor 4 (gut)
76184	103.71	48.63	-1.09	0.014843507	Down	0.000130274	Abca6	ATP-binding cassette, sub-family A (ABC1), member 6
67122	227.91	105.40	-1.11	0.017300231	Down	0.000157829	Nrarp	Notch-regulated ankyrin repeat protein
14545	323.91	148.77	-1.12	0.005492303	Down	3.71037E-05	Gdap1	ganglioside-induced differentiation-associated-protein 1
50760	66.97	30.72	-1.12	0.016366405	Down	0.000146475	Fbxo17	F-box protein 17
13170	953.84	435.62	-1.13	0.000778596	Down	3.32675E-06	Dbp	D site albumin promoter binding protein
228608	2512.41	1128.64	-1.15	8.99006E-05	Down	2.54352E-07	Smox	spermine oxidase
11864	49.60	22.02	-1.17	0.044638811	Down	0.00066072	Arnt2	aryl hydrocarbon receptor nuclear translocator 2
11555	230.91	101.81	-1.18	4.75782E-07	Down	4.94491E-10	Adrb2	adrenergic receptor, beta 2
21844	260.55	114.27	-1.19	0.020300534	Down	0.00019283	Tiam1	T cell lymphoma invasion and metastasis 1
81489	234.82	101.97	-1.20	5.76392E-07	Down	6.65618E-10	Dnajb1	DnaJ (Hsp40) homolog, subfamily B, member 1
16477	825.53	356.85	-1.21	4.46268E-06	Down	7.98793E-09	Junb	Jun-B oncogene
245026	100.74	43.50	-1.21	0.000542178	Down	2.22268E-06	Col6a6	collagen, type VI, alpha 6
218865	89.43	38.35	-1.22	0.018142954	Down	0.000166564	Chdh	choline dehydrogenase
14284	674.76	287.95	-1.23	5.06538E-12	Down	1.55236E-15	Fosl2	fos-like antigen 2
24066	185.76	79.13	-1.23	0.000180183	Down	5.82612E-07	Spry4	sprouty homolog 4 (Drosophila)
63953	753.52	319.99	-1.24	0.000644078	Down	2.7148E-06	Dusp10	dual specificity phosphatase 10
17133	95.95	40.60	-1.24	0.003563274	Down	2.28375E-05	Maff	v-maf musculoaponeurotic fibrosarcoma oncogene family, protein F (avian)
238564	17664.39	7459.98	-1.24	1.09235E-12	Down	2.5229E-16	Mylk4	myosin light chain kinase family, member 4
68149	312.89	131.84	-1.25	3.47792E-06	Down	5.62283E-09	Otub2	OTU domain, ubiquitin aldehyde binding 2
14561	89.31	37.18	-1.26	0.001394291	Down	7.25215E-06	Gdf11	growth differentiation factor 11
11910	182.39	75.26	-1.28	0.000126198	Down	3.78909E-07	Atf3	activating transcription factor 3
434215	291.41	118.61	-1.30	2.82643E-10	Down	1.14239E-13	Lrrc32	leucine rich repeat containing 32
17937	135.57	53.96	-1.33	2.49049E-06	Down	3.59503E-09	Nab2	Ngfi-A binding protein 2
229011	89.63	35.67	-1.33	0.000787127	Down	3.40866E-06	Samd10	sterile alpha motif domain containing 10
74747	399.80	157.36	-1.35	3.0776E-05	Down	7.46343E-08	Ddit4	DNA-damage-inducible transcript 4
18627	199.08	77.98	-1.35	2.63469E-06	Down	4.10743E-09	Per2	period circadian clock 2
215418	163.90	63.97	-1.36	1.6427E-06	Down	2.27639E-09	Csrnp1	cysteine-serine-rich nuclear protein 1
12227	1084.65	421.33	-1.36	4.46268E-06	Down	7.98398E-09	Btg2	B cell translocation gene 2, anti-proliferative
16658	500.97	194.57	-1.36	5.06538E-12	Down	1.75485E-15	Mafb	v-maf musculoaponeurotic fibrosarcoma oncogene family, protein B (avian)
81879	155.37	57.98	-1.42	0.002191784	Down	1.22757E-05	Tfcp2l1	transcription factor CP2-like 1
11702	13829.20	5063.81	-1.45	0.045478406	Down	0.000682741	Amd1	S-adenosylmethionine decarboxylase 1
23928	33.84	12.14	-1.48	0.025445719	Down	0.00028121	Lamc3	laminin gamma 3
114249	138.34	49.17	-1.49	0.007901088	Down	6.02196E-05	Npnt	nephronectin
170706	244.81	85.80	-1.51	0.001030789	Down	4.76143E-06	Tmem37	transmembrane protein 37
71198	914.97	319.51	-1.52	7.10016E-05	Down	1.9603E-07	Otud1	OTU domain containing 1

23882	906.31	314.89	-1.53	1.76E-14	Down	2.03245E-18	Gadd45g	growth arrest and DNA-damage-inducible 45 gamma
12029	449.58	147.47	-1.61	1.3172E-06	Down	1.67321E-09	Bcl6b	B cell CLL/lymphoma 6, member B
20855	40.91	13.14	-1.64	0.002755216	Down	1.68632E-05	Stc1	stanniocalcin 1
230810	180.19	55.90	-1.69	0.001213985	Down	6.16841E-06	Slc30a2	solute carrier family 30 (zinc transporter), member 2
229599	148.66	43.64	-1.77	9.94907E-07	Down	1.20637E-09	Gm129	predicted gene 129
12795	99.94	27.73	-1.85	5.40427E-06	Down	9.98537E-09	Plk3	polo-like kinase 3
20860	11.78	3.09	-1.93	0.049850829	Down	0.000797314	Sult1e1	sulfotransferase family 1E, member 1
381823	477.96	111.49	-2.10	2.05899E-07	Down	1.6644E-10	Apold1	apolipoprotein L domain containing 1
330723	164.37	35.49	-2.21	1.77714E-15	Down	1.02612E-19	Htra4	HtrA serine peptidase 4
18227	345.74	73.64	-2.23	9.10614E-09	Down	5.78368E-12	Nr4a2	nuclear receptor subfamily 4, group A, member 2
192663	27.19	5.23	-2.38	0.000495133	Down	1.88688E-06	Abcg4	ATP-binding cassette, sub-family G (WHITE), member 4
11838	86.50	12.31	-2.81	1.63651E-13	Down	2.83477E-17	Arc	activity regulated cytoskeletal-associated protein

Supplemental Table 4S: Immunoassay information for blood and serum chemistry, muscle adenosine triphosphate content as well as muscle and adipose tissue protein analysis.

Blood & Serum chemistry	Assay information
Bicarbonate, Ca & Pi	VetScan Comprehensive Dsiagnostic Profile, Abaxix, 500-0038
BUN	Biosource, MBS751125
25-Hydroxy vitamin D	IDS, AC-35F1
1,25-Dihydroxy Vitamin D	IDS, AC-62F1
Mouse PTH, 1-84	Immutopics, 60-2305
Creatinine	Stressgen, 907-035
Mouse Vitamin D Binding Protein	R&D Systems, DY4188-05

Muscle & adipose tissue	Assay information
ATP content	Abcam, ab83355
Collagen Assay Kit	Abcam, ab222942
Mouse IKK-alpha (phospho-Thr23) ELISA kit	LifeSpan BioSciences, LS-F1537-1
IL-1β	RayBiotech, ELM-IL1b-CL
IL-6	RayBiotech, ELM-IL6
Mouse NFKB p50 (phospho-Ser337) ELISA kit	Aviva Systems Biology, OKAG00322
Mouse total NFKB p50 ELISA kit	Mybiosource, MBS031656
Mouse NFKB p65 (phospho-Ser536) ELISA kit	RayBiotech, PEL-NFKBP65-S536-T-1
Mouse total NFKB p65 ELISA kit	RayBiotech, PEL-NFKBP65-S536-T-1
TNF	RayBiotech, ELM-TNFa
UCP1	Uscn Life Sciense, E95557Mu
UCP3	ElAab, E2068m

Supplemental Table 5S: PCR primer information.

Gene	Forward primer sequence	Reverse primer sequence	Primer Bank ID
Agt	TCTCCTTTACCACAACAAGAGCA	CTTCTCATTCACAGGGGAGGT	19705566a1
Atf3	GAGGATTTTGCTAACCTGACACC	TTGACGGTAACTGACTCCAGC	31542154a1
Atp2a2	GAGAACGCTCACACAAAGACC	CAATTCGTTGGAGCCCCAT	6806903a1
Atrogin-1	CAGCTTCGTGAGCGACCTC	GGCAGTCGAGAAGTCCAGTC	13385848a1
BMP-7	ACGGACAGGGCTTCTCCTAC	ATGGTGGTATCGAGGGTGGAA	31982487a1
Cidea	TGACATTCATGGGATTGCAGAC	GGCCAGTTGTGATGACTAAGAC	6680944a1
CD137	CGTGCAGAACTCCTGTGATAAC	GTCCACCTATGCTGGAGAAGG	20306992a1
Cox2	AACCCAGGGGATCGAGTGT	CGCAGCTCAGTGTTTGGGAT	16716411a1
Dio2	AATTATGCCTCGGAGAAGACCG	GGCAGTTGCCTAGTGAAAGGT	6753638a1
Cpt1α	CTCCGCCTGAGCCATGAAG	CACCAGTGATGATGCCATTCT	27804309a1
Csrp3	GGGGGAGGTGCAAAATGTG	CAGGCCATGCAGTGGAAACA	7304987a1
Cyfip2	ATGACCACCCACGTCACTTTG	CCTGTCCTCGAAGTTCGTGTC	19526988a1
Fhl1	GACTGCCGCAAGCCCATAA	CCAAGGGGTGAAGGCACTT	6753864a1
Fosl2	CCAGCAGAAGTTCCGGGTAG	GTAGGGATGTGAGCGTGGATA	34328117a1
Gng2	ACCGCCAGCATAGCACAAG	AGTAGGCCATCAAGTCAGCAG	6754020a1
Igf1	GTGGGGGCTCGTGTTTCTC	GATCACCGTGCAGTTTTCCA	33859572a1
IL-13Rα2	ACCGAAATGTTGATAGCGACAG	ACAATGCTCTGACAAATGCGTA	6680405a1
Itpr1	CGTTTTGAGTTTGAAGGCGTTT	CATCTTGCGCCAATTCCCG	26339686a1
Maff	AGGAGGAGGTCATCCGACTG	CTTCTCGCTCTCCAGAATGTG	23503735a1
Murf-1	GTGTGAGGTGCCTACTTGCTC	GCTCAGTCTTCTGTCCTTGGA	21523717a1
MyD88	TCATGTTCTCCATACCCTTGGT	AAACTGCGAGTGGGGTCAG	26354939a1
Myl2	ATCGACAAGAATGACCTAAGGGA	ATTTTTCACGTTCACTCGTCCT	12832296a1
MyoD	CCACTCCGGGACATAGACTTG	AAAAGCGCAGGTCTGGTGAG	6996932a1
Myostatin	AGTGGATCTAAATGAGGGCAGT	GTTTCCAGGCGCAGCTTAC	6754752a1
PAI-I	TCTGGGAAAGGGTTCACTTTACC	GACACGCCATAGGGAGAGAAG	170172561c1
Pax7	TCTCCAAGATTCTGTGCCGAT	CGGGGTTCTCTCTCTTATACTCC	34328055a1
Pgc1α	TATGGAGTGACATAGAGTGTGCT	GTCGCTACACCACTTCAATCC	238018130c1
Pgc1β	TCCTGTAAAAGCCCGGAGTAT	GCTCTGGTAGGGGCAGTGA	18875426a1
Pgf2α synthase	CTGGACTCATCGCAAACACAA	AGGAAGCCTTTGACTTCTGTCTA	6679533a1
Pparα	AGAGCCCCATCTGTCCTCTC	ACTGGTAGTCTGCAAAACCAAA	31543500a1
Pparδ	CAAGTGGGGTCAGTCATGGAA	GCTGGAAGGAAGCGTGTGTT	403943a1
Prdm16	CCCCACATTCCGCTGTGAT	CTCGCAATCCTTGCACTCA	124107622c3
Smad-3	TCTGGGAAAGGGTTCACTTTACC	GACACGCCATAGGGAGAGAAG	170172561c1
Tbx1	CTGTGGGACGAGTTCAATCAG	TTGTCATCTACGGGCACAAAG	22094109a1
TGF-β1	CTCCCGTGGCTTCTAGTGC	GCCTTAGTTTGGACAGGATCTG	6755775a1
Tlr2	GCAAACGCTGTTCTGCTCAG	AGGCGTCTCCCTCTATTGTATT	31981333a1
Tmem26	TTCCTGTTGCATTCCCTGGTC	GCCGGAGAAAGCCATTTGT	29244332a1
Tnnc1	GCGGTAGAACAGTTGACAGAG	CCAGCTCCTTGGTGCTGAT	6678369a1
Tpm3	ACCACCATCGAGGCGGTAA	CCCTTTCCTCCGCATCATCA	54912a1
Traf6	AAAGCGAGAGATTCTTTCCCTG	ACTGGGGACAATTCACTAGAGC	6678429a1
Gapdh (internal control)	AGGTCGGTGTGAACGGATTTG	TGTAGACCATGTAGTTGAGGTCA	6679937a1
